# Comprehensive investigating of MMR gene in hepatocellular carcinoma with chronic hepatitis B virus infection in Han Chinese population

**DOI:** 10.3389/fonc.2023.1124459

**Published:** 2023-03-24

**Authors:** Ning Ma, Ao Jin, Yitong Sun, Yiyao Jin, Yucheng Sun, Qian Xiao, XuanYi Sha, Fengxue Yu, Lei Yang, Wenxuan Liu, Xia Gao, Xiaolin Zhang, Lu Li

**Affiliations:** ^1^ Hebei Key Laboratory of Environment and Human Health, Department of Social Medicine and Health Care Management, School of Public Health, Hebei Medical University, Shijiazhuang, China; ^2^ Hebei Key Laboratory of Environment and Human Health, Department of Epidemiology and Statistics, School of Public Health, Hebei Medical University, Shijiazhuang, China; ^3^ Hebei Key Laboratory of Environment and Human Health, School of Basic Medicine, Hebei Medical University, Shijiazhuang, China; ^4^ The Hebei Key Laboratory of Gastroenterology, The Second Hospital of Hebei Medical University, Shijiazhuang, China

**Keywords:** HBV, HCC, SNP, MMR, haplotype, interaction

## Abstract

Hepatocellular carcinoma associated with chronic hepatitis B virus infection seriously affects human health. Present studies suggest that genetic susceptibility plays an important role in the mechanism of cancer development. Therefore, this study focused on single nucleotide polymorphisms (SNPs) of *MMR* genes associated with HBV-HCC. Five groups of participants were included in this study, which were healthy control group (HC), spontaneous clearance (SC), chronic hepatitis B group (CHB), HBV-related liver cirrhosis group (LC) and HBV-related hepatocellular carcinoma group (HBV-HCC). A total of 3128 participants met the inclusion and exclusion criteria for this study. 20 polymorphic loci on *MSH2*, *MSH3* and *MSH6* were selected for genotyping. There were four case-control studies, which were HC vs. HCC, SC vs. HCC, CHB vs. HCC and LC vs. HCC. We used Hardy-Weinberg equilibrium test, unconditional logistic regression, haplotype analysis, and gene-gene interaction for genetic analysis. Ultimately, after excluding confounding factors such as age, gender, smoking and drinking, 12 polymorphisms were found to be associated with genetic susceptibility to HCC. Haplotype analysis showed the risk haplotype GTTT (rs1805355_G, rs3776968_T, rs1428030_C, rs181747_C) was more frequent in the HCC group compared with the HC group. The GMDR analysis showed that the best interaction model was the three-factor model of *MSH2*-rs1981928, *MSH3*-rs26779 and *MSH6*-rs2348244 in SC vs. HCC group (P=0.001). In addition, we found multiplicative or additive interactions between genes in our selected SNPs. These findings provide new ideas to further explore the etiology and pathogenesis of HCC. We have attempted to explain the molecular mechanisms by which certain SNPs (*MSH2*-rs4952887, *MSH3*-rs26779, *MSH3*-rs181747 and *MSH3*-rs32950) affect genetic susceptibility to HCC from the perspectives of eQTL, TFBS, cell cycle and so on. We also explained the results of haplotypes and gene-gene interactions. These findings provide new ideas to further explore the etiology and pathogenesis of HCC.

## Introduction

Hepatocellular carcinoma (HCC), the fifth most common malignancy among worldwide, is the second leading cause of cancer-related death in China (1). Due to the insidious onset of HCC, the difficulty of early diagnosis and the rapid development of the disease, many patients have developed to advanced stage of the tumor at the time of consultation, and even after surgery or radiotherapy, the overall 5-year survival rate is less than 30%. Therefore, it is a challenge for clinicians to find out the factors that affect the susceptibility and clinical prognosis of HCC and conduct early intervention (2, 3). Although studies have suggested that different histological parameters should be used to predict the susceptibility and prognosis of HCC, a new cancer classification system (4) using molecular markers to explain the prognosis of patients with HCC holds broad prospects. Exploring the factors related to the susceptibility and prognosis of HCC can also provide new clues for finding the markers for diagnosis of HCC and the intervention targets for treatment, which has important clinical significance.

With the completion of the Human Genome Project and molecular biotechnology, gene polymorphism as an essential role of clarifying tumor susceptibility is widely concerned (5). Polymorphism is the result of mutations. Single nucleotide polymorphism (SNP), the most frequent form of human genetic variation, is a modification of a DNA sequence due to the change of a single nucleotide (6). SNPs in different locations have different functions. Missense mutation of SNPs in the exon directly alter the amino acid sequence of the encoding protein. Intron polymorphisms may have an impact on gene splicing and the degradation of messenger RNA, whereas polymorphisms in the regulatory region plays a part in regulating transcription and translation processes (7). But these SNPs work together to influence gene expression and function. Relevant studies show that SNPs in genes that regulate immunity (8–10), DNA repair (11–13), metabolism (14) are associated with genetic susceptibility to HCC. SNPs can not only affect development of disease, it can also be used as molecular genetic markers. SNPs have the obvious characteristics of high density, easy to realize high-throughput detection and stability. Convenient conditions are created for understanding the genome of individuals.

DNA repair genes are vital candidates that influences susceptibility to cancer. Repair gene defects may cause genetic instability leading to increased rates of somatic mutations. DNA mismatch repair (MMR) genes, are key factors in response to base-base mismatches and small insertion/deletions caused by misincorporation errors during DNA replication (15). Studies indicated that SNPs of specific MMR genes can affect the expression of genes, activity of enzymes and individual repair efficiency to DNA damages (16–18). As one of the most important MMR genes, *MSH2* plays a critical role in repairing mismatched DNA base. The current research indicates that *MHS2* gene polymorphism be associated with the occurrence and development of breast cancer (19), gastric cancer (20), esophageal cancer (21) and base cell carcinoma (22). M Yano (23) showed that *MSH2* closely correlate with the survival of HCC patients. However, only a few studies (24) have investigated the relationship between *MSH2* polymorphism and HCC. MSH2 works together with MSH3 or MSH6 as a heterodimer. Base–base mispairs are primarily identified by MSH2-MSH3 and large insertion/deletions are recognized by MSH2-MSH6. Both complexes perform in the repair of small insertion/deletions (25, 26). The loss of MSH2-MSH3 and/or MSH2-MSH6 expression is the most common form in MMR deficiency tumors. An integrative pan-cancer analysis show that *MSH6* mutations are closely linked to the occurrence, progression or metastasis of cancer. Moreover, *MSH6*’s high expression was linked with poor prognosis of liver cancer patients (27). In Liu’s study (28), the CT genotype of *MSH6* (rs1042821) reduced the risk of primary hepatocellular carcinoma (PHC). A recent study showed that *MSH3* leaded to microsatellie instability and promoted the occurrence and progression of HCC (29). Up to now, only a few (30) domestic reports confirmed the relationship between *MSH3*/*MSH6* gene polymorphism and HCC susceptibility, but most of the studies only stayed at the level of genetic detection.

SNPs in the same chromosomal region are not inherited randomly, but as combinations of alleles, which form haplotype blocks (31). Linkage disequilibrium (LD) defines the haplotypes of a certain population and refers to the non-random linkage of alleles at different loci (32). The genetic information provided by haplotype is more accurate and more in line with the genetic characteristics of polygenic diseases than a single allele. Besides, analysis of markers on a haplotype can reduce the complexity of analyzing SNPs in a gene or loci. Thus, haplotype is an effective tool to explore the relationship between genome and disease.

Gene-gene interaction(GxG) plays an important role in the occurrence of complex diseases. The occurrence of HCC is a complex process involving multiple genes and factors. Further exploration of GxG will help us understand the causes of population susceptibility differences and better understand the relationship between genes and diseases (33). Since studies that lack interaction and haplotype analysis lose a great deal of genetic information to better explain the molecular mechanisms that lead to individual differences in HCC susceptibility, therefore, we included the above two analyses in our study.

In conclusion, 20 SNPs on *MSH2*, *MSH3*, *MSH6* genes were selected based on candidate gene strategy. We will study the relationship between SNPs and susceptibility of HBV-related HCC from three aspects: single site genotype, multi-site haplotype and multi-site interaction. The results will provide scientific basis for screening and clinical prognosis of liver cancer susceptible population.

## Materials and methods

### Participants

Participants were collected from November 2009 to July 2016. All participants were Han Chinese in northern China, who were recruited from the First, Second and Fourth Hospitals of Hebei Medical University and the Fifth Hospital of Shijiazhuang City. The participants were divided into 5 groups: healthy control group (HC), Spontaneous clearance (SC), chronic hepatitis B group (CHB), HBV-related liver cirrhosis group (LC) and HBV-related hepatocellular carcinoma group (HBV-HCC). Healthy volunteers and HBV natural clearance patients were selected as control group in the physical examination center of the above hospitals. All the healthy controls had no history of hepatitis virus infection and liver-related diseases, their blood routine was normal and all the HBV serological markers were negative or only anti-HBs positive. SC group was defined as those with normal blood routine, normal liver function and no history of other liver diseases. Meanwhile, in their serological tests, anti-HBs and anti-HBc were positive, HBsAg, HBeAg, anti-HBe and HBV DNA were negative. CHB group: serum HBsAg or HBV DNA positive over 6 months, CHB diagnosis meets the diagnostic criteria of *China’s 2019 edition of Guidelines for Prevention and Treatment of Chronic Hepatitis B.* LC group: serum HBsAg or HBV DNA positive over 6 months, the LC diagnosis meets the *China’s 2019 Guidelines for Diagnosis and Treatment of Liver Cirrhosis*. HBV-HCC group: serum HBsAg or HBV DNA positive over 6 months, HCC diagnosis meets the *China’s 2019 edition of Guidelines for Diagnosis and Treatment of Primary Liver Cancer.*

Exclusion criteria: (i) coinfection with other viruses such as HCV, HDV and human immunodeficiency virus infection; (ii) patients with liver diseases caused by autoimmunity, alcohol and drugs; (iii) patients with acute hepatitis B and metastatic liver cancer; (iv) individuals who could not or were unwilling to participate or sign the informed consent.

To control the effects of confounding factors such as HBV and histopathological parameters and to increase the reliability of the results, we conducted four case-control studies to investigate genetic factors associated with HCC susceptibility (HC vs. HCC, SC vs. HCC, CHB vs. HCC and LC vs. HCC). Up to 24 independent variables including 20 loci and 4 other factors (age, gender, smoking and drinking, the definition of smoking and drinking status was shown in **Supplementary Table 2**) were incorporated into the Logistic Regression equation. Therefore, the sample size of each group should be at least 240.

A total of 840 healthy controls, 496 HBV natural clearance patients, 691 CHB patients, 680 LC patients and 421 HBV-HCC patients who met the criteria were included in this study. The complete personal information of the participants was collected through questionnaire survey and medical record data. All participants provided written informed consent. The procedures followed in this study were in accordance with the ethics guidelines of the 2000 Declaration of Helsinki and approved by the Ethics Committee of Hebei Medical University.

### The selection of candidate genes and SNPs

PubMed database (https://pubmed.ncbi.nlm.nih.gov/) was searched for risk-SNP in *MMR* genes associated with cancer development. SNPs located in specific regions of *MMR* genes were searched through UCSC (https://genome.ucsc.edu/), HapMap (https://www.genome.gov/) and Ensemble (https://asia.ensembl.org/index.html) databases. The function prediction of these candidate genes was performed by database GWAS4D (http://www.mulinlab.org/gwas4d/), VARAdb (http://www.licpathway.net/VARAdb/) and eQTLGen Consortium (https://eqtlgen.org/).

The selected SNPs met the following criteria: (i) The association between SNPs and HBV-related HCC had not yet been explored or needs further confirmation. (ii) The minor allele frequency (MAF) of SNP was greater than 5% in the Han population of northern China. (iii) We intended to select functional SNPs, such as enhancer, Exonic Splicing Silencer (ESS), TFBS, Exonic Splicing Enhancer (ESE) and eQTLs. Finally, a total of 20 loci were included in the study, and the details are shown in **Supplementary Table 1**.

### DNA extraction and SNP genotyping

2ml of venous blood was obtained from the participants and whole blood DNA was extracted using Genomic DNA Purification Kits (Promega, the US). NanoDrop 2000 was used to identify the concentration and purity of DNA to ensure the quality of samples. The method for SNP genotyping of all samples was based on the Sequenom Massarray platform. Primer design was performed by MassARRAY^®^ AssayDesigner3.1 combined with relevant literature. Initial multiplex PCR amplification was performed using the Sequenom amplification kit. A total of 45 cycles were performed, and the cycling conditions were set as follows: denaturation was started at 94°C for 15min, denaturation at 94°C for 20 s, annealing at 56°C for 30 s, cooling at 72°C for 60 s, and then a final extension at 72°C for 3 minutes and cooling to 4°C. At the end of the PCR reaction, the prepared SAP (shrimp alkaline phosphatase) mix solution was added to the PCR reaction plate for the alkaline phosphatase treatment reaction. Desalting was performed by reverse-phase absorption elution and MassARRAY Typer 4.0.5 was used for genotyping analysis.

In quality control, in the reaction of 384-well plates, one negative and one positive control were added in every reaction to check the reaction quality. 10% of the samples were randomly selected to repeat the data analysis.

### Statistical analysis

The SNPStats (http://bioinfo.iconcologia.net/SNPstats) was used to test the Hardy-Weinberg (H-W) balance of genotype frequencies at 20 loci. Pearson chi-square test was used to compare two or more sample rates and constituent ratios. Unconditional Logistic regression was used to analyze the association between SNPs and HCC susceptibility (co-dominant, dominant, recessive), and calculated the odds ratio (OR) at 95% CI. The above statistical analyses were carried out in SPSS V26.0 software (IBM, Armonk, New York). Haplovie4.2 was used to establish the structure of haplotype blocks and perform haplotype analysis. The software GMDR (v9.0) was used to determine the best interaction model of 20 SNPs in each case-control group. The gene-gene multiplicative interaction was evaluated using Logistic regression model in SPSS V26.0. The additive interaction was performed using SPSS26.0 and Excel 2019 with written program. The following three indices: Synergy index (*S*), Attributable Proportion of interaction (*AP*) and Relative Excess Risk of Interaction (*RERI*), are used to evaluate the results of the additive model. When *S*=1, there is no interaction between the factors and the disease; when *S*>1, there is a positive interaction, which means that the pathogenic effect is stronger when the two factors are present together than the effect of the two factors alone; if *S*<1, there is a negative interaction between the two factors. *AP* indicates the proportion of the interaction effect in the combined effect of two exposure factors.*RERI* was used to describe the magnitude of risk attributable to interaction. The larger the absolute value of *RERI*, the stronger the interaction between the factors. In the present study, additive interaction was considered to exist when the confidence intervals for *RERI* and *AP* did not contain “ 0 “ and the confidence interval for *S* did not contain “1”.

## Results

### Participants characteristics

There were statistically significant differences in age, gender, smoking and alcohol consumption among the four case-control studies (*P*<0.001) (**Supplementary Table 2**). The Hardy-Weinberg Equilibrium results showed that all 20 loci conformed to the H-W equilibrium law (*P*>0.05), which indicated that the studied sample was representative of the population. Details were shown in **Supplementary Table 3**. Distribution of genotypes and minor allele frequencies of the 20 SNPs among the five groups was shown in **Supplementary Table 4**.

### The results in HC vs. HCC

The HC group was used as the control group and the HCC group as the case group to study the influence of genetic factors on the susceptibility to HCC. As shown in **Supplementary Table 5**, univariate analysis revealed that *MSH3*-rs32950 (GG), *MSH3*-rs181747 (CC), *MSH3*-rs863221 (GG), *MSH3*-rs1042821(GA) and *MSH3*-rs1042821(GA/AA) were risk factors. *MSH2*-rs3776968 (T), *MSH2*-rs3776968 (CT), *MSH2*-rs3776968 (CT/TT), *MSH3*-rs33002 (AT), *MSH3*-rs33008(GC) and *MSH3*-rs33008(GC/CC) were protective factors.

Using multiple logistic regression analysis, we found that in the co-dominant model, *MSH6*-rs1042821 (GA) was a protective genotype compared with AA (*P*=0.014, OR=0.688). In the dominant model, only one loci entered the equation, *MSH6*-rs2348244 (TC+CC) was a risk factor compared with TT, which was more likely to develop into HCC (*P*=0.037, OR=1.371). In addition, rs33008 (CC) and rs863221 (GG) of *MSH3* were significantly associated with increased risk of HCC in recessive model (*P*=0.023, OR=1.959 and *P*=0.003, OR=1.867). Details were provided in **Table 1**.

**Table 1:** Multiple logistic regression analysis of predictive factors for hepatocellular carcinoma between HC and HCC in three models.

Variable	B	S.E	Wald	*P*	OR(95%CI)
Codominant					
age	1.025	0.113	82.013	1.35×10^-19^*	2.787(2.233,3.480)
gender (Male)	0.451	0.171	6.968	0.008*	1.569(1.123,2.192)
smoke (Yes)	1.341	0.183	53.543	2.53×10^-13^*	3.823(2.669,5.475)
drink (Yes)	0.475	0.184	6.676	0.010*	1.608(1.122,2.306)
* MSH3*-rs2112416			10.122	0.006*	
* MSH3*-rs2112416(TA)	-0.381	0.154	6.118	0.013*	0.683(0.505,0.924)
* MSH3*-rs2112416(AA)	0.203	0.212	0.916	0.338	1.226(0.808,1.858)
* MSH6*-rs1042821			8.170	0.017*	
* MSH6*-rs1042821(GA)	-0.374	0.152	6.066	0.014*	0.688(0.511,0.926)
* MSH6*-rs1042821(AA)	0.360	0.343	1.103	0.294	1.433(0.732,2.805)
Dominant					
age	1.013	0.112	81.147	2.10×10^-19^*	2.753(2.209,3.431)
gender (Male)	0.438	0.170	6.657	0.010*	1.549(1.111,2.160)
smoke (Yes)	1.344	0.182	54.787	1.34×10^-13^*	3.834(2.686,5.473)
drink (Yes)	0.480	0.182	6.953	0.008*	1.617(1.131,2.311)
* MSH6*-rs2348244(TC+CC)	0.316	0.151	4.342	0.037*	1.371(1.019,1.845)
Recessive					
age	1.031	0.113	82.504	1.05×10^-19^*	2.803(2.244,3.501)
gender (Male)	0.434	0.171	6.465	0.011*	1.543(1.105,2.156)
smoke (Yes)	1.366	0.184	55.119	1.13×10^-13^*	3.918(2.732,5.619)
drink (Yes)	0.462	0.184	6.296	0.012*	1.587(1.106,2.277)
* MSH3*-rs2112416(AA)	0.545	0.202	7.259	0.007*	1.725(1.160,2.564)
* MSH3*-rs33008(CC)	0.672	0.296	5.169	0.023*	1.959(1.097,3.498)
* MSH3*-rs863221(GG)	0.624	0.207	9.049	0.003*	1.867(1.243,2.803)

HC, health control; HCC, hepatocellular carcinoma; *:P<0.05.

**Figure 1:**
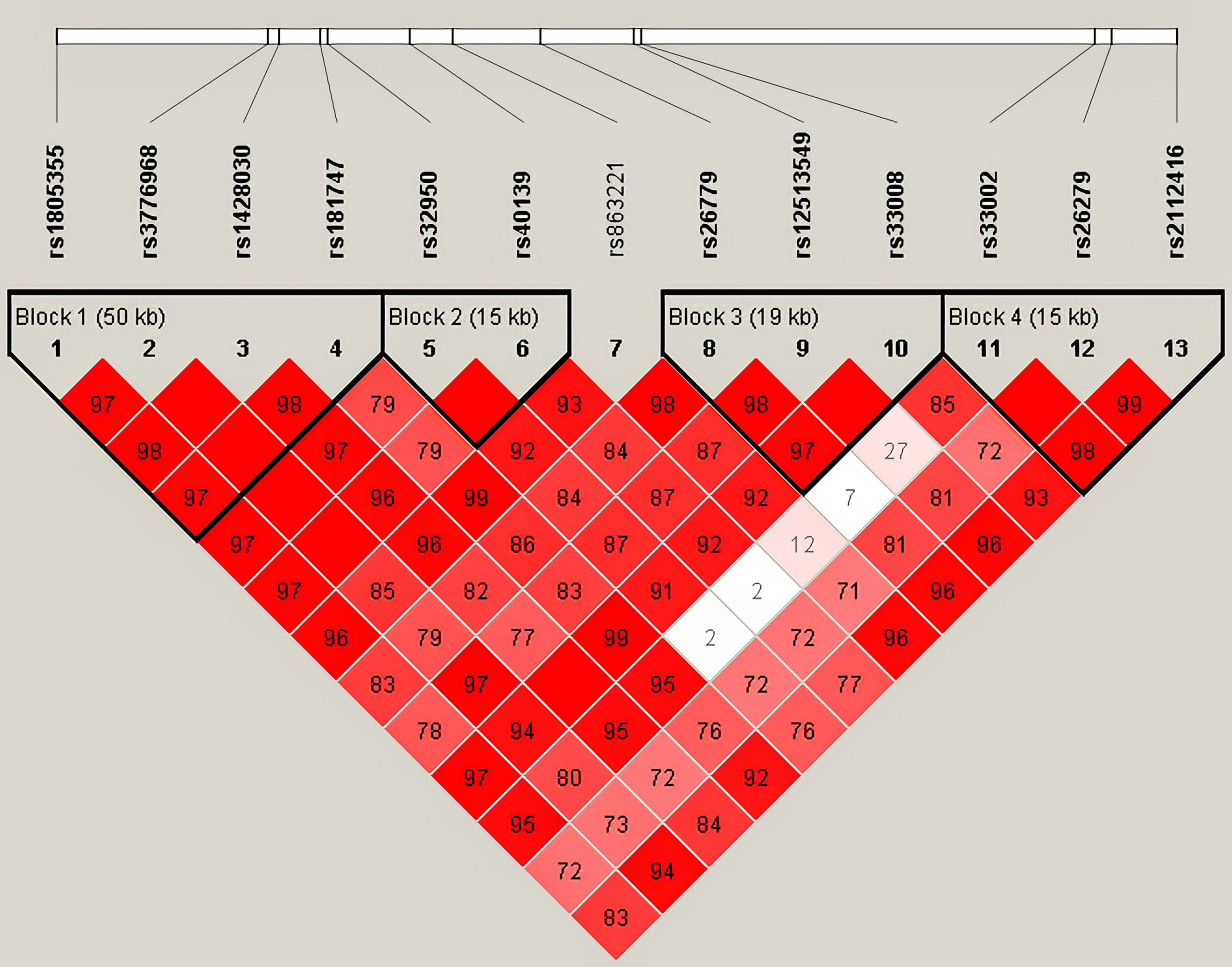
Haplotype analysis for 13 SNPs between HC and HCC by Haploview 4.2.

Haplotype analysis for 13 SNPs in *MSH3* gene between HC and HCC groups was shown in **Table 2** (**Figure 1**). Thirteen loci in *MSH3* formed four haplotype blocks. Block 1 consisted of rs1805355_G, rs3776968_T, rs1428030_C, rs181747_C and the four haplotypes accounted for 99.1% of the distribution. The distribution of haploid GTTT was statistically significant in HC vs. HCC (*P*=0.0187, OR=0.797), and it was a protective factor for HCC. Block 2 was composed of rs32950_G and rs40139_G, and the haplotype formed accounts for 100% of the population distribution. However, haplotype GG and AA had no statistically significant effect on HCC susceptibility. Block 3, including CGG, TTG, CGC and TGG, accounted for 99.5%. Rs26779_T, rs12513549_T, rs33008_C were in almost absolute LD. CGC significantly decreased the risk of HCC (*P*=0.0410, OR=0.818). Furthermore, Block 4 formed by rs33002_T-rs26279_A-rs2112416_A. The number of individuals carrying the AAT haplotype (*P*=0.0268, OR=0.352) was more in the HC group. Haplotype analysis of 7 SNPs in the *MSH2* and *MSH6* genes between the HC and HCC groups was not statistically significant (**Supplementary Table 6**).

**Table 2:** Haplotype analysis for 13 SNPs in *MSH3* gene between HC and HCC groups by Haploview 4.2.

Haplotype	Freq.	Case, Control Ratios	*P* value	OR (95%CI)
Block 1				
ACCC	0.358	311.0: 527.0, 588.0: 1088.0	0.3172	1.092(0.919,1.297)
GCTT	0.324	288.9: 549.1, 526.2: 1149.8	0.1191	1.150(0.964,1.371)
GTTT	0.278	207.9: 630.1, 490.5: 1185.5	0.0187*	0.797(0.660,0.963)
GCTC	0.031	27.1: 810.9, 50.3: 1625.7	0.7537	1.083(0.673,1.742)
Block 2				
GG	0.646	525.0: 313.0, 1092.0: 574.0	0.1526	0.882(0.742,1.048)
AA	0.354	313.0: 525.0, 574.0: 1092.0	0.1526	1.134(0.954,1.348)
Block 3				
CGG	0.375	330.2: 509.8, 611.9: 1060.1	0.1858	1.121(0.945,1.329)
TTG	0.272	234.5: 605.5, 448.1: 1223.9	0.5520	1.059(0.880,1.275)
CGC	0.256	193.8: 646.2, 448.7: 1223.3	0.0410*	0.818(0.674,0.993)
TGG	0.092	77.6: 762.4, 154.4: 1517.6	0.9989	1.009(0.758,1.343)
Block 4				
TAT	0.390	317.5: 522.5, 662.7: 1013.3	0.3987	0.929(0.784,1.102)
AAA	0.365	316.9: 523.1, 600.7: 1075.3	0.3560	1.084(0.913,1.287)
AGT	0.228	199.4: 640.6, 374.1: 1301.9	0.4257	1.081(0.888,1.315)
AAT	0.013	5.2: 834.8, 28.3: 1647.7	0.0268*	0.352(0.136,0.916)

HC, healthy control; HCC, hepatocellular carcinoma; *: P<0.05.

Block1, including:rs1805355_G, rs3776968_T, rs1428030_C, rs181747_C, were in LD.

Block 2, including:rs32950_G, rs40139_G, were in LD.

Block 3, including: rs26779_T, rs12513549_T, rs33008_C, were in LD.

Block 4, including:rs33002_T, rs26279_A, rs2112416_A, were in LD.

**Figure 2:**
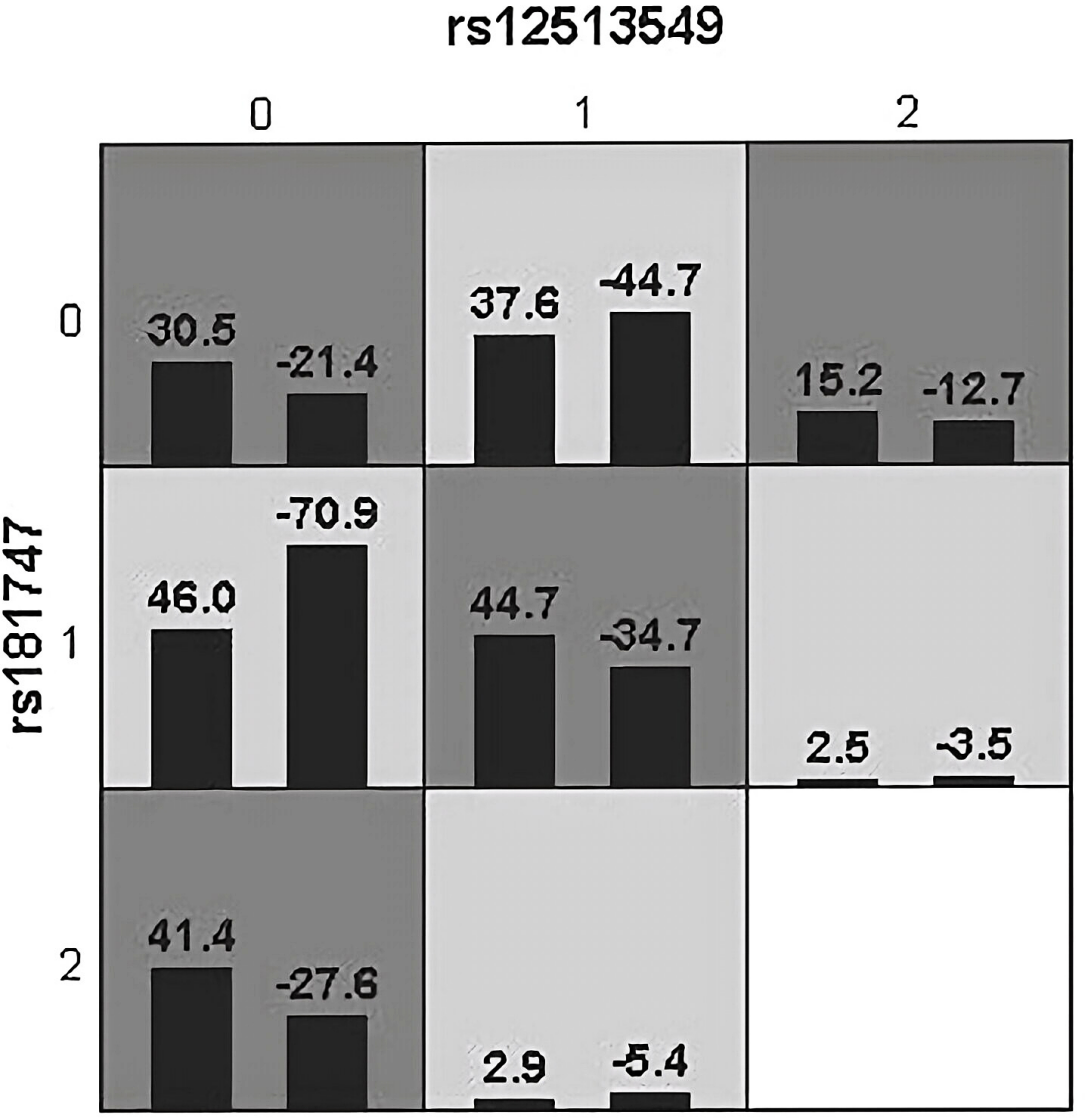
Epistatic interaction for HBV chronic hepatic diseases involving rs12513549 and rs181747 between HCC and HC group. rs12513549(0, 1, 2)=rs12513549(GG, GT, TT);rs181747(0, 1, 2)=rs181747(TT, TC, CC). The scores of HCC group (left black bar in boxes) and HC (right black bar in boxes) are shown for each genotype combination.

We used GMDR to analyze gene-gene interactions at 20 loci. The results showed that the 2-factor model constituted by *MSH3*-rs12513549 and *MSH3*-rs181747 was the best interaction model related to HCC susceptibility. According to the 2-factor interaction combination, the study subjects were redivided into the high-risk group and the low-risk group (**Table 3**). The morbidity of high-risk group was 1.931 times higher than that of low-risk group (**Figure 2**). The results of multiplicative interaction analysis showed that *MSH6*-rs2348244 (TC+CC) and *MSH3*-rs3776968 (CC) had positive multiplicative interaction, and both of them jointly led to the occurrence of the disease (*P*<0.001, OR=1.701) (**Table 4**). The interaction based on additive model did not show significant statistical significance (**Supplementary Table 7**).

**Table 3:** GMDR analysis of 20 loci in HC vs. HCC, SC vs. HCC, CHB vs. HCC, and LC vs HCC groups.

Group	Model	TBA	Sign Test(P)	CVC
HC vs. HCC				
	rs2112416	0.4886	0.6230	3/10
	rs12513549 rs181747^#^	0.5696	0.0010*	9/10
	rs2303428 rs33002 rs2348244	0.5129	0.3770	4/10
	rs2303428 rs26779 rs1042821 rs2348244	0.5390	0.1719	3/10
	rs2303428 rs26779 rs181747 rs1042821 rs2348244	0.5472	0.1719	4/10
SC vs. HCC				
	rs1981928	0.5419	0.3770	9/10
	rs13019654 rs26279	0.5613	0.0107*	7/10
	rs1981928 rs26779 rs2348244^#^	0.6140	0.0010*	8/10
	rs1981928 rs26779 rs33008 rs2348244	0.5938	0.0547	4/10
	rs13019654 rs2303428 rs33002 rs32950 rs2348244	0.5633	0.0107	3/10
CHB vs. HCC				
	rs863221	0.5567	0.0010*	10/10
	rs26779 rs1042821	0.5434	0.0547	6/10
	rs13019654 rs26779 rs1042821	0.5431	0.1719	6/10
	rs13019654 rs26779 rs33002 rs1042821	0.5295	0.3770	6/10
	rs13019654 rs2303428 rs26779 rs33002 rs2348244	0.5227	0.1719	6/10
LC vs. HCC				
	rs2112416	0.5360	0.0547	9/10
	rs26779 rs1042821	0.5020	0.3770	3/10
	rs1981928 rs863221 rs2348244	0.4857	0.6230	4/10
	rs1981928 rs33002 rs863221 rs2348244	0.4755	0.8281	5/10
	rs1981928 rs13019654 rs33002 rs863221 rs2348244	0.4764	0.9453	3/10

TBA, testing balanced accuracy; CVC, cross-validation consistency; The limit dimension was set to 5; #, the best model;Confounding factors such as gender, age and the history of drinking and smoking were controlled in the operation. *: P<0.05.

**Table 4:** Multiplicative interaction analysis of SNPs in HC vs. HCC, SC vs. HCC, CHB vs. HCC, LC vs. HCC groups by logistic regression.

Group	Variable	B	S.E	Wald	*P*	OR (95%CI)
HC vs. HCC						
	age	1.054	0.113	87.647	7.83×10-^21^*	2.87(2.301,3.578)
	gender (Male)	0.448	0.168	7.100	0.008*	1.565(1.126,2.176)
	drink (Yes)	0.458	0.183	6.276	0.012*	1.581(1.105,2.263)
	smoke (Yes)	1.322	0.182	52.791	3.71×10-^13^*	3.752(2.626,5.360)
	rs2348244* rs3776968	0.531	0.146	13.259	2.71×10^-4^*	1.701(1.278,2.264)
SC vs. HCC						
	age	0.903	0.123	53.617	2.44×10^-13^*	2.467(1.937,3.142)
	gender (Male)	0.404	0.183	4.855	0.028*	1.498(1.046,2.147)
	drink (Yes)	0.882	0.223	15.617	7.80×10^-5^*	2.415(1.56,3.740)
	smoke (Yes)	0.730	0.222	10.817	0.001*	2.075(1.343,3.205)
	rs40139* rs4952887	0.466	0.180	6.674	0.010*	1.593(1.119,2.267)
	rs13019654* rs2348244	0.505	0.168	9.026	0.003*	1.657(1.192,2.303)
	rs2303428* rs26779	0.530	0.238	4.947	0.026*	1.699(1.065,2.710)
	rs1042821* rs33002	1.599	0.515	9.623	0.002*	4.948(1.802,13.587)
CHB vs. HCC						
	age	1.437	0.120	143.212	5.28×10^-33^*	4.208(3.325,5.324)
	gender (Male)	0.424	0.184	5.319	0.021*	1.528(1.066,2.189)
	smoke (Yes)	0.664	0.158	17.624	2.70×10^-5^*	1.942(1.425,2.648)
	rs1981928 * rs26779	0.495	0.179	7.667	0.006*	1.641(1.156,2.331)
	rs1042821* rs1981928	-0.584	0.183	10.151	0.001*	0.558(0.389,0.799)
	rs1042821*rs32950	0.684	0.318	4.617	0.032*	1.981(1.062,3.696)
LC vs. HCC						
	age	0.752	0.120	39.164	3.90×10^-10^*	2.122(1.676,2.685)
	drink(Yes)	0.476	0.177	7.251	0.007*	1.61(1.138,2.277)
	smoke (Yes)	0.369	0.175	4.435	0.035*	1.446(1.026,2.038)
	rs181747* rs4952887	0.573	0.214	7.166	0.007*	1.773(1.166,2.697)
	rs2303428* rs26779	0.590	0.242	5.932	0.015*	1.804(1.122,2.900)

HC, healthy control; SC, spontaneous clearance; CHB, chronic hepatitis B; LC, liver cirrhosis; HCC, hepatocellular carcinoma; Genotype assignment based on the optimum risk association results of genetic models: HC vs. HCC: rs2348244: TT (0), TC+CC (1); rs3776968: CT+TT (0), CC (1); SC vs. HCC: rs40139: AA+AG (0), GG (1); rs4952887: CT+TT (0), CC (1); rs13019654: TT (0), GG+GT (1); rs2348244: TT (0), TC+CC (1); rs2303428: CC (0), TC+TT (1); rs26779: TT (1), CT+CC (0); rs1042821: GG+GA (0), AA(1); rs33002: AA (0), AT+TT (1); CHB vs. HCC: rs1981928: AA (0), TA+TT (1); rs26779: CT+TT (0), CC (1); rs1042821: GG (0), GA+AA (1); rs32950: AG+GG (0), AA (1); LC vs. HCC: rs181747: TC+TT (0), CC (1); rs4952887: CT+TT (0), CC (1); rs2303428: TT (0), TC+CC (1); rs26779: CT+CC (0), TT (1). *: P<0.05.

### The results in SC vs HCC

In this group of analysis to explore the susceptibility loci of HCC, we used SC as the control group and HCC as the case group. As shown in **Supplementary Table 5**, univariate analysis revealed *MSH6*-rs1042821 (AA) and *MSH2*-rs1981928 (TA) were risk factors. *MSH6*-rs1042821(AA) was a protective factor.

Multiple logistic regression analysis showed that the individuals carrying *MSH2*-rs1981928 (TA) genotype were more likely to have increased risk of HCC in co-dominant (*P*=0.001, OR=2.182). In the same way, *MSH6*-rs1981928 (TA+AA) was shown to be associated with an increased risk of HCC occurrence in the dominant model (*P*=0.012, OR=1.738). In the recessive model, four loci were statistically significant. The *MSH3*-rs3776968 (TT) reduced the risk of HCC (*P*=0.006, OR=0.347). *MSH3*-rs33002 (TT), *MSH3*-rs26779 (TT), *MSH3*-rs32950 (GG) were more likely to increase HCC risk (*P*=0.038, OR=1.746; *P*=0.045, OR=1.603 and *P*=0.001, OR=1.943). Details were provided in **Table 5**.

**Table 5:** Multiple logistic regression analysis of predictive factors for hepatocellular carcinoma between SC and HCC in three models.

Variable	B	S.E	Wald	*P*	OR (95%CI)
Codominant					
age	0.958	0.128	55.687	8.50×10-^14^*	2.608(2.027,3.354)
gender (Male)	0.528	0.186	8.072	0.004*	1.696(1.178,2.443)
smoke (Yes)	0.724	0.226	10.247	0.001*	2.062(1.324,3.211)
drink (Yes)	0.923	0.227	16.596	4.60×10^-5^*	2.517(1.615,3.925)
* MSH2*-rs1981928			11.814	0.003*	
* MSH2*-rs1981928(TA)	0.780	0.238	10.778	0.001*	2.182(1.370,3.478)
* MSH2*-rs1981928(AA)	0.409	0.240	2.914	0.088	1.505(0.941,2.407)
* MSH3*-rs32950			7.461	0.024*	
* MSH3*-rs32950(AG)	-0.387	0.235	2.710	0.100	0.679(0.428,1.077)
* MSH3*-rs32950(GG)	0.073	0.239	0.092	0.762	1.075(0.673,1.718)
Dominant					
age	0.948	0.127	55.513	9.28×10^-14^*	2.580(2.011,3.311)
gender (Male)	0.495	0.183	7.305	0.007*	1.641(1.146,2.349)
smoke (Yes)	0.666	0.223	8.913	0.003*	1.947(1.257,3.016)
drink (Yes)	0.933	0.225	17.215	3.30×10^-5^*	2.542(1.636,3.950)
* MSH6*-rs1981928(TA+AA)	0.553	0.220	6.318	0.012*	1.738(1.129,2.674)
Recessive					
age	0.961	0.129	55.86	7.78×10^-14^*	2.614(2.032,3.364)
gender (Male)	0.544	0.187	8.473	0.004*	1.722(1.194,2.483)
smoke (Yes)	0.763	0.227	11.258	0.001*	2.145(1.373,3.350)
drink (Yes)	0.880	0.227	14.991	1.08×10^-4^*	2.411(1.544,3.765)
* MSH2*-rs13019654(TT)	-0.615	0.323	3.625	0.057	0.541(0.287,1.018)
* MSH3*-rs26779(TT)	0.472	0.235	4.036	0.045*	1.603(1.012,2.540)
* MSH3*-rs33002(TT)	0.557	0.268	4.309	0.038*	1.746(1.032,2.954)
* MSH3*-rs32950(GG)	0.664	0.191	12.092	0.001*	1.943(1.336,2.826)
* MSH3*-rs3776968(TT)	-1.059	0.384	7.592	0.006*	0.347(0.163,0.737)

SC, spontaneous clearance; HCC, hepatocellular carcinoma; *: P<0.05.

**Figure 3:**
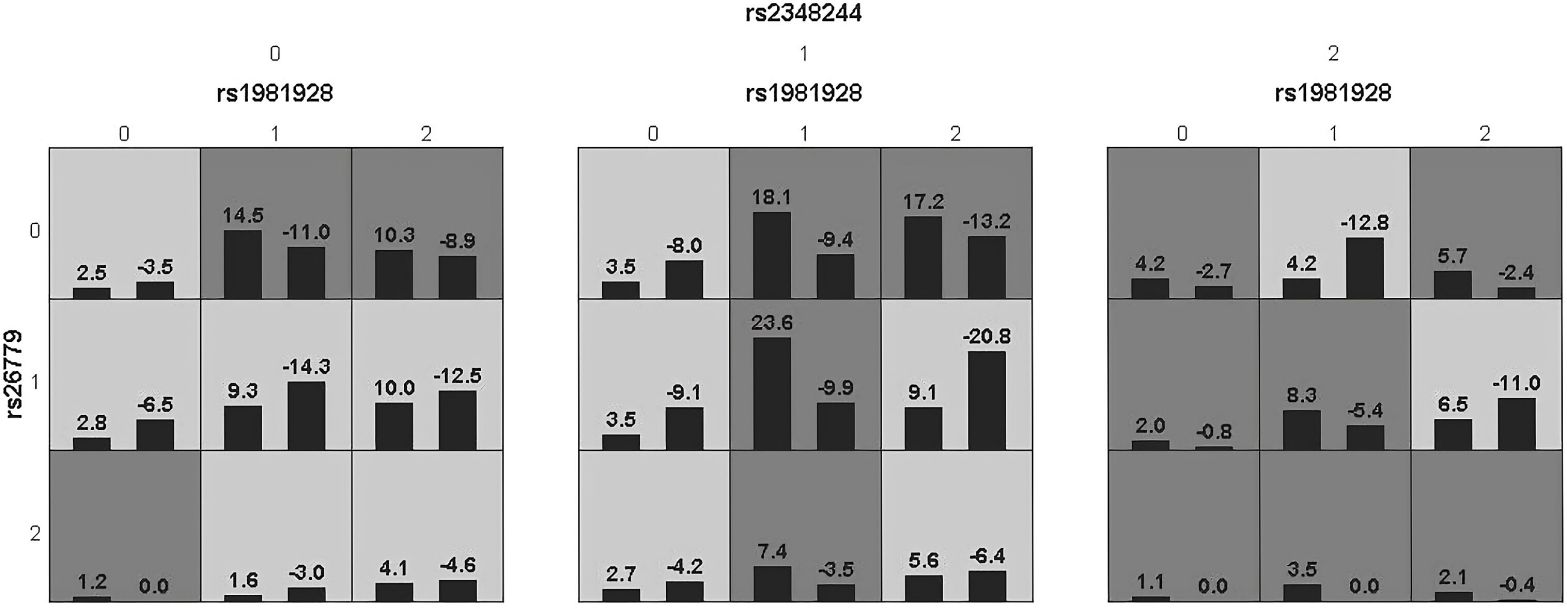
Epistatic interaction for HCC involving rs1981928, rs26779, rs2348244 between HCC and SC group.Rs1981928(0,1,2)=rs1981928 (TT, TA, AA); rs26779(0,1,2)=rs26779 (CC, CT, TT); rs2348244(0,1,2)=rs2348244 (TT,TC,CC).The scores of HCC group (left black bar in boxes) and SC group (right black bar in boxes) are shown for each genotype combination.

Haplotype analysis showed that linkage disequilibrium existed at 20 candidate loci on *MSH2*, *MSH3* and *MSH6*, However, there was no difference in the distribution of these haplotypes in the SC and HCC groups (**Supplementary Tables 6**, **8**). **Table 3** summarized the results obtained from GMDR analysis. The best interaction model was the three-factor model of *MSH2*-rs1981928, *MSH3*-rs26779 and *MSH6*-rs2348244 (*P*=0.001). The cross-validation consistency (CVC) was 8/10, and the balance accuracy was 61.40%. According to the model, the study subjects were redivided into the high-risk group and the low-risk group. The morbidity of high-risk group was 3.150 (2.059, 4.819) times higher than that of low-risk group (**Figure 3**).

**Table 4** showed the results of the multiplicative interaction analysis. There were four groups with statistically significant results, *MSH3*-rs40139 (GG) and *MSH2*-rs4952887 (CC) (*P*=0.010, OR=1.593), *MSH2*-13019654 (GG+GT) and *MSH6*-2348244 (TC+CC) (*P*=0.003, OR=1.657), *MSH2*-rs2303428 (TC+TT) and *MSH3*-rs26779 (TT) (*P*=0.026, OR=1.699), *MSH3*-1042821 (AA) and *MSH3*-33002 (AT+TT) (*P*=0.002, OR=4.948). All four groups of loci with risk genotypes had synergistic effects in increasing HCC susceptibility.

The results of the additive model suggested that only *MSH2*-rs1981928 and *MSH3*-rs40139 existed additive interactions (**Table 6**; **Supplementary Table 9**). The risk genotype TT/TA of *MSH2*-rs1981928 and the risk genotype GG of *MSH3*-rs40139 were correlated with the occurrence of the HCC when exposed alone (*P*=0.025, OR=1.726 and *P*=0.002, OR=2.355), and the two genotypes were also correlated with the occurrence of the HCC when exposed together (*P*=0.047, OR=1.744). Since the relative excess risk of interaction was -1.339 [*RERI*=-1.339(-2.503, -0.174)], there was a negative additive interaction. When the two factors existed together, the risk of occurrence was 0.357 times of that when the two factors existed alone [*S*=0.357(0.168, 0.758)].

**Table 6:** The positive results of additive interaction analysis of SNPs and four other factors in SC vs. HCC group.

SNP1	SNP2	SC	HCC	B	*P*	OR (95%CI)	RERI/AP/S
rs1981928	rs40139						
AA	AA+AG	140	95			1	
AA	GG	68	68	0.794	0.006	2.212(1.257,3.894)	**RERI:-1.249(-2.355,-0.143)**
TT+TA	AA+AG	160	151	0.519	0.034	1.680(1.040,2.713)	**AP:-0.760(-1.462,-0.057)**
TT+TA	GG	116	104	0.497	0.081	1.644(0.940,2.874)	**S:0.340(0.151,0.766)**

SC, spontaneous clearance; HCC, hepatocellular carcinoma; RERI, Relative Excess Risk of Interaction; AP, Attributable Proportion of interaction; S, Synergy index. When calculating covariance matrix, take SNPs other than the analysis SNPs, together with gender, age, the history of drinking and smoking as control variables.The bold font shows statistical significance.

### The results in CHB vs. HCC

When we took the CHB group as the control group and the HCC group as the case group, the results of the univariate analysis (**Supplementary Table 5**) showed that *MSH6*-rs1042821 (AA), *MSH3*-rs1428030 (CC), *MSH3*-rs1805355 (AA), *MSH3*-rs181747 (CC) and *MSH6*-rs1042821(AA) were risk factors. *MSH2*-rs4952887 (CT), *MSH3*-rs863221 (GT) and *MSH3*-rs26779 (CT) were protective factors.

Multiple logistic regression analysis showed that in the co-dominant model, individuals carrying *MSH3*-rs26779 (CT) had a lower risk of the development of HCC than those carrying CC (*P*=0.007, OR=0.650). In the dominant model, the genotype *MSH3*-rs26779 (CT+TT) was protective factors for cancerization (*P*=0.008, OR=0.655). Details were provided in **Table 7**.

**Table 7:** Multiple logistic regression analysis of predictive factors for hepatocellular carcinoma between CHB and HCC in three models.

Variable	B	S.E	Wald	*P*	OR (95%CI)
Codominant					
age	1.417	0.120	139.017	4.37×10^-32^*	4.125(3.259,5.220)
gender (Male)	0.459	0.185	6.118	0.013*	1.582(1.100,2.275)
smoke (Yes)	0.687	0.159	18.739	1.50×10^-5^*	1.988(1.457,2.714)
* MSH3*-rs26779			8.609	0.014*	
* MSH3*-rs26779(CT)	-0.431	0.161	7.193	0.007*	0.650(0.474,0.890)
* MSH3*-rs26779(TT)	0.006	0.211	0.001	0.979	1.006(0.664,1.522)
Dominant					
age	1.412	0.120	138.04	7.14×10^-32^*	4.104(3.243,5.194)
gender (Male)	0.462	0.185	6.223	0.013*	1.588(1.104,2.283)
smoke (Yes)	0.700	0.159	19.474	1.00×10^-5^*	2.014(1.476,2.748)
* MSH3*-rs2112416(TA+AA)	-0.341	0.157	4.707	0.030*	0.711(0.523,0.968)
* MSH3*-rs26779(CT+TT)	-0.423	0.158	7.116	0.008*	0.655(0.480,0.894)
Recessive					
age	1.403	0.120	137.832	7.93×10^-32^*	4.068(3.219,5.142)
gender (Male)	0.475	0.184	6.650	0.010*	1.608(1.121,2.307)
smoke (Yes)	0.681	0.158	18.623	1.60×10^-5^*	1.976(1.450,2.693)

CHB, chronic hepatitis B; HCC, hepatocellular carcinoma; *:P <0.05.

The linkage disequilibrium existed at 20 candidate loci on *MSH2*, *MSH3* and *MSH6*, which was shown by Haplotype analysis. But there was no difference in the distribution of these haplotypes in the CHB and HCC groups (**Supplementary Tables 6**, **8**). GMDR analysis did not reveal the best interaction model (**Table 3**). The multiplication interaction showed that there was a positive multiplication interaction between *MSH2*-rs1981928 (TA+TT) and *MSH3*-rs26779 (CC) (*P*=0.006, OR=1.641). There was a positive multiplication interaction between *MSH6*-rs1042821 (GA+AA) and *MSH3*-rs32950 (AA) (*P*=0.032, OR=1.981). There was a negative multiplicative interaction between *MSH6*-rs1042821 (GA+AA) and *MSH2*-rs1981928 (TA+TT) (*P*=0.001, OR=0.558) (**Table 4**). No statistical significance was found in the additive interaction analysis. (**Supplementary Table 10**).

### The results in LC vs. HCC

With the LC group as the control group and the HCC group as the case group, the results of the univariate analysis (**Supplementary Table 5**) showed that *MSH3*-rs181747 (TT) and *MSH3*-rs181747 (CC) were risk factors. *MSH2*-rs4952887 (T), *MSH2*-rs4952887 (CT) and *MSH2*-rs4952887(CT/TT) were protective factors.

**Table 8** showed the results of the multiple logistic regression analysis. In the co-dominant models, *MSH3*-rs181747 (CC) can increase the risk of HCC (*P*=0.005, OR=3.759). In the recessive model, *MSH3*-rs1428030 (CC) was a protective factor (*P*=0.046, OR=0.326). Both *MSH3*-rs26779 (TT) and *MSH3*-rs181747 (CC) were risk factors for the development of HCC (*P*=0.035, OR=1.485, and *P*=0.004, OR=4.686).

**Table 8:** Multiple logistic regression analysis of predictive factors for hepatocellular carcinoma between LC and HCC in three models.

Variable	B	S.E	Wald	*P*	OR (95%CI)
Codominant					
age	0.806	0.125	41.703	1.06×10^-10^*	2.239(1.753,2.860)
gender (Male)	0.349	0.175	3.969	0.046*	1.418(1.006,2.000)
smoke (Yes)	0.368	0.179	4.236	0.040*	1.444(1.018,2.049)
drink (Yes)	0.396	0.184	4.643	0.031*	1.486(1.036,2.130)
* MSH3*-rs2112416			8.659	0.013*	
* MSH3*-rs2112416(TA)	-0.879	0.303	8.433	0.004*	0.415(0.229,0.751)
* MSH3*-rs2112416(AA)	-1.030	0.478	4.641	0.031*	0.357(0.140,0.911)
* MSH3*-rs181747			7.772	0.021*	
* MSH3*-rs181747(TC)	0.568	0.303	3.511	0.061	1.765(0.974,3.197)
* MSH3*-rs181747(CC)	1.324	0.476	7.741	0.005*	3.759(1.479,9.552)
Dominant					
age	0.742	0.121	37.563	8.85×10^-10^*	2.100(1.657,2.663)
smoke (Yes)	0.407	0.175	5.373	0.020*	1.502(1.065,2.118)
drink (Yes)	0.496	0.177	7.822	0.005*	1.641(1.160,2.323)
Recessive					
age	0.768	0.122	39.295	3.64×10^-10^*	2.155(1.695,2.740)
smoke (Yes)	0.410	0.177	5.365	0.021*	1.506(1.065,2.131)
drink (Yes)	0.454	0.179	6.444	0.011*	1.575(1.109,2.236)
* MSH3*-rs1428030(CC)	-1.122	0.562	3.981	0.046*	0.326(0.108,0.980)
* MSH3*-rs26779(TT)	0.395	0.187	4.449	0.035*	1.485(1.028,2.143)
* MSH3*-rs181747(CC)	1.545	0.544	8.075	0.004*	4.686(1.615,13.598)

LC, liver cirrhosis; HCC, hepatocellular carcinoma; *:P<0.05.

Haplotype analysis revealed the linkage disequilibrium at 20 candidate loci on *MSH2*, *MSH3* and *MSH6*, nevertheless, the distribution of these haplotypes did not differ between the LC and HCC groups. (**Supplementary Tables 6**, **8**). GMDR analysis did not reveal the best interaction model of multi-genes between LC and HCC (**Table 3**). The result of the multiplicative interaction analysis was shown in **Table 4**, and the risk of HCC attributable to the interaction of *MSH3*-rs181747 (CC) and *MSH2*-rs4952887 (CC) was 1.773 (*P*=0.007, OR=1.773). The risk of HCC attributable to the interaction of *MSH2*-rs2303428 (TC+CC) and *MSH3*-rs26779 (TT) was 1.804 (*P*=0.015, OR=1.804). The risk genotypes of the two pairs of loci mentioned above acted synergistically in causing the development of HCC, resulting in an additional increase in the likelihood of HCC development. There were no statistically significant results found in the additive interaction analysis of SNPs between LC and HCC (**Supplementary Table 11**).

## Discussion

For *MSH2*-rs4952887, univariate analysis showed that CHB patients carrying CT genotype were less likely to develop HCC and that T served as a protective base to reduce the risk of HCC development in LC group. The eQTLGen Consortium (https://eqtlgen.org/) showed that rs4952887 as a cis-eQTL affected the expression of *MSH2*. The MSH2 protein, protects DNA from AID-induced somatic mutations (34). Activation-induced cytidine deaminase (AID) is a well-defined molecule capable of inducing mutations in human DNA sequences (35). Experimental data from Tadayuki Kou et al. confirmed that AID may promote the production of *TP53* mutations as well as other possible somatic mutations, leading to the development of HCC in the context of chronic liver disease (36). When *MSH2* expression was defective, the incidence of tumor increased in mice with AID expression. Our results showed that the rs4952887 T base was much less distributed in the HCC group, and also that this locus acted as an eQTL affecting *MSH2* expression. Therefore, we speculate that the rs4952887 T base upregulates *MSH2* expression, leading to a reduced rate of AID-induced somatic mutations and hence reducing the risk of HCC development.

*MSH3*-rs26779 TT was a risk factor for HCC in SC vs. HCC and CHB vs. HCC. Both VARAdb (http://www.licpathway.net/VARAdb/) and 3DSNP (http://www.omic.tech/3dsnpv2/) show that rs26779 affects the binding of MYC transcription factor. The transcription factor MYC is encoded by the proto-oncogene *MYC (* 37). It is one of the most common oncogenic transcriptional regulators, affecting almost all cellular processes (38), and is important for the control of cell growth and viability (39). Xia P and Zhang H et al. demonstrated that the transcription factor MYC promoted *WDR4* transcription by binding to the *WDR4* promoter region in hepatocellular carcinoma cells (40). WDR4 has a wide range of effects on the cell cycle and the immune infiltration of hepatocellular carcinoma cells (41, 42). Existing studies had shown that WDR4 enhanced the translation of *CCNB1* by promoting the binding of *CCNB1* mRNA to EIF2A, and CCNB1 is a key molecule regulating G2/M phase progression. This pathway can affect tumor growth and metastasis by affecting the cell cycle (40). In addition, CCNB1 can also affect the stability of P53 by promoting the ubiquitination of P53, thereby promoting the occurrence of HCC (40). This is consistent with the results of our study. Because the mutant genotype TT has a high distribution frequency in HCC patients. Therefore, we hypothesized that *MSH3*-rs26779 T may be related to the development of HCC by affecting the affinity of MYC transcription factor.

*MSH3*-rs181747 CC was a risk factor for HCC susceptibility according to the results of our study. According to the database SNP2TFBS (https://ccg.epfl.ch/snp2tfbs/), *MSH3*-rs181747 may affect the binding of PRRX2 transcription factor. PRRX2 transcription factor was confirmed to regulate *IL-6* transcription in HCC cells (43). IL-6, as a tumor promoting cytokine, triggers the Janus kinase (JAK) associated with the receptor, stimulating STAT3 phosphorylation and activation. It participates in the processes of anti-apoptosis, angiogenesis, proliferation, invasion of cancer cells, and is related to the occurrence of cervical cancer, prostate cancer and colorectal cancer (44–47). In addition, IL-6 secretion accelerates the migration of macrophages and neutrophils in the liver, which amplifies the inflammatory response and the development of tumours (48, 49). In consequence, we hypothesize that the rs181747 CC genotype affects the binding of PRRX2 to IL-6 and that the generated IL-6 contributes to cancer development and progression *via* p-STAT3 and facilitates chemotactic movement of immune cells.

Our results show that *MSH3*-rs32950 GG is a risk factor for HCC susceptibility. From the “motif change” of VARAdb (http://www.licpathway.net/VARAdb/), *MSH3*-rs32950 A>G polymorphism affects the motif sequence of FOXP1 transcription factor, resulting in changes in affinity between FOXP1 and transcription factor binding sites. Forkhead box P1 (FOXP1) is a member of a family of wing-helix transcription factors (50). FOXP1 is expressed in a variety of human cancer tissues (51–53). It has been demonstrated that silencing FOXP1 significantly inhibits the proliferation of hepatocellular carcinoma cells *in vitro* and *in vivo*. Further studies demonstrated that the mechanism by which downregulation of FOXP1 inhibits HCC cell proliferation is the induction of G1/S phase cell cycle arrest. This process may be related to the dysregulation of retinoblastoma protein (Rb) (54). Rb is a tumor suppressor and is essential for the cell cycle and the negative regulation of tumor progression. Dephosphorylated Rb is associated with cell cycle G1/S phase arrest and thus plays an anti-oncogenic role (55). FOXP1 has been experimentally shown to be a transcriptional repressor of Rb (56). FOXP1 interferes with cell cycle G1/S phase arrest by downregulating dephosphorylated Rb, thereby dysregulating the cell cycle and contributing to cancer development. In addition, it has been shown that FOXP1 can affect the activation of the TGF-β pathway by binding to the transcription factors Smad2 and Smad3, thereby causing CD8^+^ T cells de-lymphotoxicity in hepatocellular carcinoma tissues. T cells unresponsiveness causes T cells anti-tumor failure, which may also contributes to the development of HCC (57, 58). In summary, we suggest that rs32950 GG may influence HCC development by regulating the transcription factor FOXP1.

Haplotypes are combinations of alleles on multiple locus that are co-inherited on the same chromosome. For multigene or multilocus diseases, certain associations may have to be represented by a haplotype of multiple loci rather than a single locus. Certain haplotypes are presented in clusters in the population at a higher frequency, which is called linkage disequilibrium. In this study, we found that the risk haplotype GTTT (rs1805355_G, rs3776968_T, rs1428030_C, rs181747_C) was more frequent in the HCC group compared with the HC group. In the database Ensembl (https://asia.ensembl.org/index.html), we found that there was linkage disequilibrium among *MSH3*-rs1805355, *MSH3*-rs3776968, *MSH3*-rs1428030 and *MSH3*-rs181747. Although the results of the haplotype analysis currently lack a biological mechanism to explain them, this finding strongly supports the credibility of our findings.

Gene-gene interactions are characterized by the combined effects of two or more genes on the phenotype that differ from their independent effects (59). It is considered to be one of the fundamental inheritance patterns of complex diseases. Therefore, our study used three interaction analyses to further explore the association between gene-gene interaction and genetic susceptibility to HCC. For example, rs181747 not only formed the best interaction model with rs12513549 in GMDR analysis, but also had a multiplicative interaction with rs4952887. In addition, rs26779 not only formed the best three-factor interaction model with rs2348244 and rs1981928 in GMDR analysis, but also had multiplication interaction with rs2303428 and rs1981928, respectively. Besides, rs1981928 also had multiplication interaction with rs1042821 and additive interaction with rs40139. We have statistically demonstrated that there were interactions between these loci, and these factors work together to influence people’s risk of developing HCC. This provides clues to the mechanistic study of genetic susceptibility to HCC.

Our study has some advantages. For most of the candidate SNPs, this was the first time to study their relationship with HCC. Second, our sample size was large enough to increase the power of the test. Then, we used multi-angle statistical methods to confirm the warning effect of candidate SNPs on HCC susceptibility, which may provide new clues for the establishment of HCC susceptibility or prognosis model and even exploring HCC diagnostic markers. Finally, as candidate SNPs influence the tumour immune microenvironment, they may be expected to provide targets for intervention in immunotherapy. And exploring the potential of new therapies that target the tumour microenvironment and induce immune activation in the treatment of HCC will be an interesting avenue for future research in this field (48). However, our study has several limitations. Firstly, all samples included in this study were from Hebei Province, China. Future studies with a larger sample size and multiple centers are needed to confirm the results of this study. Besides, we were not able to integrate the potential effect of the HBV vaccination history in our study, as such, our results may be biased and should be interpreted with caution. Then, our research design is a retrospective case-control study and the findings may provide clues to predict HCC susceptibility and prognosis but cannot verify the causal relationship. The next step is a follow-up study, that is, to collect more risk factors affecting the susceptibility and prognosis of HCC for the sample population (including environmental factors and genetic factors), and follow up the prognosis of HCC patients to establish a clinical prediction model for HCC susceptibility and prognosis.The pathogenic mechanism of SNPs located in the gene regulatory region can be studied by designing molecular biology experiments from the perspectives of eQTL, TFBS or promoter.

## Data availability statement

The datasets presented in this study can be found in online repositories. The names of the repository/repositories and accession number(s) can be found below: https://figshare.com/articles/dataset/Genotyping_data_xlsx/21707537.

## Ethics statement

The procedures followed in this study were in accordance with the ethics guidelines of the 2000 Declaration of Helsinki and approved by the Ethics Committee of Hebei Medical University. All participants provided written informed consent.

## Author contributions

Concept and design: NM, XZ and LL; Writing of article: NM, AJ, YTS and YJ; Statistical analysis: NM, AJ, QX, XS, and YTS; Specimen collection and questionnaire investigation: FY, LY, XG, LL, YCS; Inputting data: FY, XZ and WL; SNP sample management: AJ, YTS and YJ; Experiments and procedures: NM, WL and XZ; Financial support: NM and XZ. All authors contributed to the article and approved the submitted version.
