# Supplementary material for: Comprehensive investigating of MMR gene in hepatocellular carcinoma with chronic hepatitis B virus infection in Han Chinese population
Source: Front Oncol. 2023 Mar 24;13:1124459. doi: 10.3389/fonc.2023.1124459 (PMC10079871; doi:10.3389/fonc.2023.1124459)
Supplement: Supplementary file 1 [file DataSheet_1.docx]

***Supplementary Material***

**1 SUPPLEMENTARY TABLES**

| Supplementary table 1 :The basic information of selected SNPs from Ensembl. | | | | | |
| --- | --- | --- | --- | --- | --- |
| Gene | SNP | Location in Gene Region | Function Prediction | MAF | Alleles |
| *MSH2* | rs1981928 | intron variant | eQTL/Risk-SNP/enhancer | 0.383 | T＞A |
| *MSH2* | rs4952887 | intron variant | eQTL/Risk-SNP | 0.194 | C＞T |
| *MSH2* | rs13019654 | intron variant | eQTL/Risk-SNP/enhancer | 0.291 | G＞T |
| *MSH2* | rs2303428 | splice region variant | eQTL/Risk-SNP/enhancer | 0.262 | T＞C |
| *MSH2* | rs12999145 | intron variant | eQTL/Risk-SNP/enhancer | 0.461 | A＞G |
| *MSH3* | rs1428030 | intron variant | eQTL/Risk-SNP/enhancer | 0.296 | T＞C |
| *MSH3* | rs2112416 | regulatory region variant | eQTL/enhancer | 0.330 | T＞A |
| *MSH3* | rs26279 | missense variant | eQTL/Splicing(ESE or ESS)/nsSNP/Risk-SNP/enhancer | 0.335 | G＞A |
| *MSH3* | rs26779 | intron variant | eQTL/Risk-SNP/enhancer | 0.466 | C＞T |
| *MSH3* | rs33002 | intron variant | eQTL/Risk-SNP/enhancer | 0.316 | A＞T |
| *MSH3* | rs33008 | intron variant | eQTL/Risk-SNP/enhancer | 0.218 | G＞C |
| *MSH3* | rs12513549 | intron variant | eQTL/Risk-SNP/enhancer | 0.369 | G＞T |
| *MSH3* | rs1805355 | synonymous variant | eQTL/Risk-SNP/enhancer | 0.291 | G＞A |
| *MSH3* | rs181747 | intron variant | eQTL/Risk-SNP | 0.325 | T＞C |
| *MSH3* | rs32950 | intron variant | eQTL/Risk-SNP | 0.466 | A＞G |
| *MSH3* | rs863221 | non coding transcript exon variant | eQTL/TFBS/Risk-SNP/super-enhancer/enhancer | 0.442 | T＞G |
| *MSH3* | rs3776968 | intron variant | eQTL/Risk-SNP/enhancer | 0.238 | C＞T |
| *MSH3* | rs40139 | intron variant | eQTL/TFBS/Risk-SNP/super-enhancer/enhancer | 0.466 | A＞G |
| *MSH6* | rs1042821 | missense variant | eQTL/TFBS/nsSNP/Risk-SNP/super-enhancer/enhancer | 0.218 | G＞A |
| *MSH6* | rs2348244 | intron variant | eQTL/Risk-SNP/enhancer | 0.466 | T＞C |
| Abbreviations: MAF, minor allele frequency; ESE, Exonic Splicing Enhancer; ESS, Exonic Splicing Silencer; TFBS, transcription factor binding sites; eQTL, expression quantitative trait locus; SNPs, single nucleotide polymorphisms. Ensemble data are from the Han Chinese in Beijing, China. | | | | | |

| Supplementary table 2 :The baseline data of study subjects. | | | | | | | | | | | | | |
| --- | --- | --- | --- | --- | --- | --- | --- | --- | --- | --- | --- | --- | --- |
| Factors | HC | NC | CHB | LC | HCC | HC vs HCC | | NC vs HCC | | CHB vs HCC | | LC vs HCC | |
|  | N=840 | N=496 | N=691 | N=680 | N=421 |  |  |  |  |  |  |  |  |
|  | n(%) | n(%) | n(%) | n(%) | n(%) | χ^2^ | *P* | χ^2^ | *P* | χ^2^ | *P* | χ^2^ | *P* |
| Age |  |  |  |  |  | 18.223 | 2.00×10^-5^* | 13.958 | 1.87×10^-4^* | 60.897 | 6.01×10^-15^* | 23.021 | 2.00×10^-6^* |
| ≤60 | 628(74.8) | 370(74.6) | 579(83.8) | 521(76.6) | 266(63.2) |  |  |  |  |  |  |  |  |
| >60 | 212(25.2) | 126(25.4) | 112(16.2) | 159(23.4) | 155(36.8) |  |  |  |  |  |  |  |  |
| Gender |  |  |  |  |  | 65.968 | 4.58×10^-16^* | 37.872 | 7.55×10^-10^* | 13.227 | 2.76×10^-4^* | 9.383 | 0.002* |
| Male | 454(54.0) | 290(58.5) | 466(67.4) | 470(69.1) | 326(77.6) |  |  |  |  |  |  |  |  |
| Female | 386(46.0) | 206(41.5) | 225(32.6) | 210(30.9) | 94(22.4) |  |  |  |  |  |  |  |  |
| Drink |  |  |  |  |  | 97.809 | 4.61×10^-23^* | 93.228 | 4.66×10^-22^* | 21.309 | 4.00×10^-6^* | 32.654 | 1.10×10^-8^* |
| N | 559(66.5) | 343(69.2) | 356(51.5) | 374(55.0) | 157(37.3) |  |  |  |  |  |  |  |  |
| Y | 281(33.5) | 153(30.8) | 335(48.5) | 306(45.0) | 264(62.7) |  |  |  |  |  |  |  |  |
| Smoke |  |  |  |  |  | 174.985 | 6.03×10^-40^* | 93.949 | 3.24×10^-22^* | 50.738 | 1.06×10^-12^* | 31.886 | 1.63×10^-8^* |
| N | 661(78.7) | 362(73.0) | 437(63.2) | 400(58.8) | 174(41.3) |  |  |  | 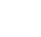 |  |  |  |  |
| Y | 179(21.3) | 134(27.0) | 254(36.8) | 280(41.2) | 247(58.7) |  |  |  |  |  |  |  |  |
| Abbreviations: HC, healthy control; NC, natural clearance; CHB, chronic hepatitis B; LC, liver cirrhosis; HCC, hepatocellular carcinoma; *: *P*＜0.05. Drinking: the person consumes rice wine, beer, liquor or wine at least once a week on average continuously or cumulatively for 1 year or more. Smoking: the person smokes at least one cigarette a day on average for 1 year or more continuously or cumulatively. | | | | | | | | | | | | | |

| Supplementary table 3: The test of Hardy-Weinberg Equilibrium for the 20 selected SNPs in all individuals. | | | | | |
| --- | --- | --- | --- | --- | --- |
| SNP | Genotype | Number | MAF | χ^2^ | *p* |
| *MSH2*-rs1981928 | TT | 1197 | 0.378 | 0.006 | 0.939 |
|  | TA | 1459 |  |  |  |
|  | AA | 442 |  |  |  |
| *MSH2*-rs4952887 | CC | 2224 | 0.152 | 2.022 | 0.155 |
|  | TC | 824 |  |  |  |
|  | TT | 62 |  |  |  |
| *MSH2*-rs13019654 | GG | 1815 | 0.237 | 0.906 | 0.341 |
|  | GT | 1100 |  |  |  |
|  | TT | 183 |  |  |  |
| *MSH2*-rs2303428 | TT | 1373 | 0.329 | 3.724 | 0.054 |
|  | TC | 1417 |  |  |  |
|  | CC | 312 |  |  |  |
| *MSH2*-rs12999145 | AA | 836 | 0.474 | 3.709 | 0.054 |
|  | AG | 1610 |  |  |  |
|  | GG | 675 |  |  |  |
| *MSH3*-rs1428030 | TT | 1287 | 0.359 | 1.763 | 0.184 |
|  | TC | 1388 |  |  |  |
|  | CC | 415 |  |  |  |
| *MSH3*-rs2112416 | TT | 1207 | 0.374 | 0.200 | 0.655 |
|  | AT | 1459 |  |  |  |
|  | AA | 1426 |  |  |  |
| *MSH3*-rs26279 | GG | 1778 | 0.242 | 1.864 | 0.172 |
|  | GA | 1170 |  |  |  |
|  | AA | 168 |  |  |  |
| *MSH3*-rs26779 | CC | 1225 | 0.373 | 0.022 | 0.882 |
|  | TC | 1450 |  |  |  |
|  | TT | 434 |  |  |  |
| *MSH3*-rs33002 | AA | 1180 | 0.380 | 1.317 | 0.251 |
|  | AT | 1497 |  |  |  |
|  | TT | 435 |  |  |  |
| *MSH3*-rs33008 | GG | 1761 | 0.250 | 3.046 | 0.081 |
|  | GC | 1123 |  |  |  |
|  | CC | 211 |  |  |  |
| *MSH3*-rs12513549 | GG | 1580 | 0.287 | 0.380 | 0.537 |
|  | GT | 1255 |  |  |  |
|  | TT | 263 |  |  |  |
| *MSH3*-rs1805355 | GG | 1268 | 0.359 | 0.015 | 0.903 |
|  | AG | 1414 |  |  |  |
|  | AA | 398 |  |  |  |
| *MSH3*-rs181747 | TT | 1165 | 0.386 | 0.001 | 0.975 |
|  | TC | 1469 |  |  |  |
|  | CC | 462 |  |  |  |
| *MSH3*-rs32950 | AA | 1226 | 0.368 | 0.950 | 0.330 |
|  | AG | 1390 |  |  |  |
|  | GG | 425 |  |  |  |
| *MSH3*-rs863221 | TT | 1302 | 0.352 | 1.087 | 0.297 |
|  | GT | 1373 |  |  |  |
|  | GG | 393 |  |  |  |
| *MSH3*-rs3776968 | CC | 1662 | 0.270 | 0.509 | 0.476 |
|  | TC | 1205 |  |  |  |
|  | TT | 233 |  |  |  |
| *MSH3*-rs40139 | AA | 1250 | 0.367 | 0.886 | 0.347 |
|  | AG | 1414 |  |  |  |
|  | GG | 430 |  |  |  |
| *MSH6*-rs1042821 | GG | 1871 | 0.217 | 3.342 | 0.068 |
|  | AG | 1078 |  |  |  |
|  | AA | 127 |  |  |  |
| *MSH6*-rs2348244 | TT | 1028 | 0.428 | 1.074 | 0.300 |
|  | TC | 1488 |  |  |  |
|  | CC | 581 |  |  |  |
| Abbreviations: MAF, minor allele frequency; SNPs, single nucleotide polymorphisms. | | | | | |

| Supplementary table 4: Distribution of genotypes and minor allele frequencies of the 20 SNPs among the five groups. | | | | | |
| --- | --- | --- | --- | --- | --- |
| Genotype | HC | NC | CHB | LC | HCC |
| *MSH2*-rs1981928 |  |  |  |  |  |
| TT | 105(12.5%) | 82(16.5%) | 101(14.6%) | 103(15.1%) | 51(12.1%) |
| TA | 415(49.4%) | 194(39.1%) | 327(47.3%) | 318(46.8%) | 205(48.7%) |
| AA | 313(37.3%) | 214(43.1%) | 255(36.9%) | 251(36.9%) | 164(39.0%) |
| A | 1041 | 622 | 837 | 820 | 533 |
| T | 625 | 358 | 529 | 524 | 307 |
| MAF(T allele) | 0.37515 | 0.365306 | 0.387262 | 0.389881 | 0.365476 |
| *MSH2*-rs4952887 |  |  |  |  |  |
| CC | 617(73.5%) | 350(70.6%) | 476(68.9%) | 466(68.5%) | 315(74.8%) |
| CT | 214(25.5%) | 130(26.2%) | 192(27.8%) | 193(28.4%) | 95(22.6%) |
| TT | 7(0.8%) | 14(2.8%) | 14(2.0%) | 18(2.6%) | 9(2.1%) |
| C | 1448 | 830 | 1144 | 1125 | 725 |
| T | 228 | 158 | 220 | 229 | 113 |
| MAF(T allele) | 0.136038 | 0.159919 | 0.16129 | 0.169129 | 0.134845 |
| *MSH2*-rs13019654 |  |  |  |  |  |
| GG | 472(56.2%) | 304(61.3%) | 405(58.6%) | 387(56.9%) | 247(58.7%) |
| GT | 316(37.6%) | 148(29.8%) | 246(35.6%) | 242(35.6%) | 148(35.2%) |
| TT | 50(6.0%) | 42(8.5%) | 30(4.3%) | 39(5.7%) | 22(5.2%) |
| G | 1260 | 756 | 1056 | 1016 | 642 |
| T | 416 | 232 | 306 | 320 | 192 |
| MAF(T allele) | 0.24821 | 0.234818 | 0.22467 | 0.239521 | 0.230216 |
| *MSH2*-rs2303428 |  |  |  |  |  |
| TT | 366(43.6%) | 216(43.5%) | 293(42.4%) | 316(46.5%) | 182(43.2%) |
| TC | 392(46.7%) | 214(43.1%) | 319(46.2%) | 298(43.8%) | 194(46.1%) |
| CC | 80(9.5%) | 58(11.7%) | 71(10.3%) | 62(9.1%) | 41(9.7%) |
| T | 1124 | 646 | 905 | 930 | 558 |
| C | 552 | 330 | 461 | 422 | 276 |
| MAF(C allele) | 0.329356 | 0.338115 | 0.337482 | 0.31213 | 0.330935 |
| *MSH2*-rs12999145 |  |  |  |  |  |
| AA | 209(24.9%) | 138(27.8%) | 182(26.3%) | 191(28.1%) | 116(27.6%) |
| AG | 455(54.2%) | 234(47.2%) | 360(52.1%) | 351(51.6%) | 210(49.9%) |
| GG | 176(21.0%) | 124(25.0%) | 143(20.7%) | 137(20.1%) | 95(22.6%) |
| A | 873 | 510 | 724 | 733 | 442 |
| G | 807 | 482 | 646 | 625 | 400 |
| MAF(G allele) | 0.480357 | 0.485887 | 0.471533 | 0.460236 | 0.475059 |
| *MSH3*-rs1428030 |  |  |  |  |  |
| TT | 351(41.8%) | 210(42.3%) | 277(40.1%) | 274(40.3%) | 175(41.6%) |
| TC | 378(45.0%) | 198(39.9%) | 321(46.5%) | 314(46.2%) | 177(42.0%) |
| CC | 109(13.0%) | 74(14.9%) | 81(11.7%) | 83(12.2%) | 68(16.2%) |
| T | 1080 | 618 | 875 | 862 | 527 |
| C | 596 | 346 | 483 | 480 | 313 |
| MAF(C allele) | 0.355609 | 0.358921 | 0.35567 | 0.357675 | 0.372619 |
| *MSH3*-rs2112416 |  |  |  |  |  |
| TT | 330(39.3%) | 186(37.5%) | 257(37.2%) | 257(37.8%) | 177(42.0%) |
| TA | 398(47.4%) | 230(46.4%) | 334(48.3%) | 329(48.4%) | 168(39.9%) |
| AA | 105(12.5%) | 72(14.5%) | 87(12.6%) | 88(12.9%) | 74(17.6%) |
| T | 1508 | 602 | 848 | 843 | 522 |
| A | 608 | 374 | 508 | 505 | 316 |
| MAF(A allele) | 0.364946 | 0.383197 | 0.374631 | 0.374629 | 0.377088 |
| *MSH3*-rs26279 |  |  |  |  |  |
| GG | 36(4.3%) | 28(5.6%) | 46(6.7%) | 36(5.3%) | 22(5.2%) |
| GA | 304(36.2%) | 182(36.7%) | 257(37.2%) | 270(39.7%) | 157(37.3%) |
| AA | 500(59.5%) | 286(57.7%) | 380(55.0%) | 372(54.7%) | 240(57.0%) |
| A | 1304 | 754 | 1017 | 1014 | 637 |
| G | 376 | 238 | 349 | 342 | 201 |
| MAF(G allele) | 0.22381 | 0.239919 | 0.25549 | 0.252212 | 0.239857 |
| *MSH3*-rs26779 |  |  |  |  |  |
| CC | 340(40.5%) | 198(39.9%) | 249(36.0%) | 263(38.7%) | 175(41.6%) |
| CT | 388(46.2%) | 234(47.2%) | 334(48.3%) | 324(47.6%) | 170(40.4%) |
| TT | 110(13.1%) | 64(12.9%) | 99(14.3%) | 90(13.2%) | 71(16.9%) |
| C | 1068 | 630 | 832 | 850 | 520 |
| T | 608 | 362 | 532 | 504 | 312 |
| MAF(T allele) | 0.362768 | 0.364919 | 0.390029 | 0.37223 | 0.375 |
| *MSH3*-rs33002 |  |  |  |  |  |
| AA | 286(34.0%) | 198(39.9%) | 265(38.4%) | 265(39.0%) | 166(39.4%) |
| TA | 435(51.8%) | 226(45.6%) | 328(47.5%) | 319(46.9%) | 189(44.9%) |
| TT | 119(14.2%) | 72(14.5%) | 87(12.6%) | 93(13.7%) | 64(15.2%) |
| A | 1007 | 622 | 858 | 849 | 521 |
| T | 673 | 370 | 502 | 505 | 317 |
| MAF(T allele) | 0.400595 | 0.372984 | 0.369118 | 0.372969 | 0.378282 |
| *MSH3*-rs33008 |  |  |  |  |  |
| GG | 435(51.8%) | 290(58.5%) | 411(59.5%) | 375(55.1%) | 250(59.4%) |
| GC | 347(41.3%) | 160(32.3%) | 218(31.5%) | 256(37.6%) | 142(33.7%) |
| CC | 51(6.1%) | 40(8.1%) | 50(7.2%) | 43(6.3%) | 27(6.4%) |
| G | 1217 | 740 | 1040 | 1006 | 642 |
| C | 449 | 240 | 318 | 342 | 196 |
| MAF(C allele) | 0.269508 | 0.244898 | 0.234168 | 0.253709 | 0.23389 |
| *MSH3*-rs12513549 |  |  |  |  |  |
| GG | 446(53.1%) | 248(50.0%) | 333(48.2%) | 334(49.1%) | 219(52.0%) |
| GT | 320(38.1%) | 204(41.1%) | 280(40.5%) | 286(42.1%) | 165(39.2%) |
| TT | 66(7.9%) | 36(7.3%) | 71(10.3%) | 55(8.1%) | 35(8.3%) |
| G | 1212 | 700 | 946 | 954 | 603 |
| T | 452 | 276 | 422 | 396 | 235 |
| MAF(T allele) | 0.271635 | 0.282787 | 0.30848 | 0.293333 | 0.28043 |
| *MSH3*-rs1805355 |  |  |  |  |  |
| GG | 341(40.6%) | 212(42.7%) | 277(40.1%) | 269(39.6%) | 169(40.1%) |
| GA | 387(46.1%) | 212(42.7%) | 321(46.5%) | 315(46.3%) | 179(42.5%) |
| AA | 108(12.9%) | 66(13.3%) | 77(11.1%) | 82(12.1%) | 65(15.4%) |
| G | 1069 | 636 | 875 | 853 | 517 |
| A | 603 | 344 | 475 | 479 | 309 |
| MAF(A allele) | 0.360646 | 0.35102 | 0.351852 | 0.35961 | 0.374092 |
| *MSH3*-rs181747 |  |  |  |  |  |
| TT | 318(37.9%) | 186(37.5%) | 248(35.9%) | 252(37.1%) | 161(38.2%) |
| TC | 397(47.3%) | 228(46.0%) | 335(48.5%) | 334(49.1%) | 175(41.6%) |
| CC | 118(14.0%) | 78(15.7%) | 97(14.0%) | 87(12.8%) | 82(19.5%) |
| T | 1033 | 600 | 831 | 838 | 497 |
| C | 633 | 384 | 529 | 508 | 339 |
| MAF(C allele) | 0.379952 | 0.390244 | 0.388971 | 0.377415 | 0.405502 |
| *MSH3*-rs32950 |  |  |  |  |  |
| AA | 98(11.7%) | 68(13.7%) | 107(15.5%) | 85(12.5%) | 67(15.9%) |
| AG | 372(44.3%) | 226(45.6%) | 317(45.9%) | 303(44.6%) | 172(40.9％) |
| GG | 361(43.0%) | 184(37.1%) | 247(35.7%) | 266(39.1%) | 168(39.9%) |
| G | 1094 | 594 | 811 | 835 | 508 |
| A | 568 | 362 | 531 | 473 | 306 |
| MAF(A allele) | 0.341757 | 0.378661 | 0.395678 | 0.361621 | 0.375921 |
| *MSH3*-rs863221 |  |  |  |  |  |
| TT | 373(44.4%) | 200(40.3%) | 266(38.5%) | 278(40.9%) | 185(43.9%) |
| TG | 362(43.1%) | 218(44.0%) | 321(46.5%) | 306(45.0%) | 166(39.4％) |
| GG | 96(11.4%) | 62(12.5%) | 89(12.9%) | 81(11.9%) | 65(15.4%) |
| T | 1108 | 618 | 853 | 862 | 536 |
| G | 554 | 342 | 499 | 468 | 296 |
| MAF(G allele) | 0.333333 | 0.35625 | 0.369083 | 0.35188 | 0.355769 |
| *MSH3*-rs3776968 |  |  |  |  |  |
| CC | 400(47.6%) | 282(56.9%) | 396(57.3%) | 346(50.9%) | 238(56.5%) |
| CT | 377(44.9%) | 162(32.7%) | 236(34.2%) | 279(41.0%) | 151(35.9％) |
| TT | 59(7.0%) | 44(8.9%) | 52(7.5%) | 48(7.1%) | 30(7.1%) |
| C | 1177 | 726 | 1028 | 971 | 627 |
| T | 495 | 250 | 340 | 375 | 211 |
| MAF(T allele) | 0.296053 | 0.256148 | 0.248538 | 0.278603 | 0.25179 |
| *MSH3*-rs40139 |  |  |  |  |  |
| AA | 104（12.4%） | 66（13.3%） | 107（15.5%） | 86（12.6%） | 67（15.9%） |
| AG | 368（43.8%） | 236（47.6） | 318（46.0%） | 313（46.0%） | 179（42.5%） |
| GG | 361（43.0%） | 186（37.5%） | 257（37.2%） | 273（40.1%） | 173（41.1%） |
| G | 1090 | 608 | 832 | 859 | 525 |
| A | 576 | 368 | 532 | 485 | 313 |
| MAF(A allele） | 0.345738 | 0.377049 | 0.390029 | 0.360863 | 0.373508 |
| *MSH6*-rs1042821 |  |  |  |  |  |
| GG | 461（54.9%） | 306（61.7%） | 418（60.5%） | 427（62.8%） | 259（61.5%） |
| GA | 328（39.0%） | 170（34.3%） | 237（34.3%） | 209（30.7%） | 134（31.8%） |
| AA | 33（3.9%） | 14（2.8%） | 22（3.2%） | 33（4.9%） | 25（5.9%） |
| G | 1250 | 782 | 1073 | 1063 | 652 |
| A | 394 | 198 | 281 | 275 | 184 |
| MAF(A allele） | 0.239659 | 0.202041 | 0.207533 | 0.205531 | 0.220096 |
| *MSH6*-rs2348244 |  |  |  |  |  |
| TT | 288（34.3%） | 170（34.3%） | 219（31.7%） | 219（32.2%） | 132（31.4%） |
| TC | 391（46.5%） | 232（46.8%） | 331（47.9%） | 330（48.5%） | 204（48.5%） |
| CC | 155（18.5%） | 92（18.5%） | 128（18.5%） | 125（18.4%） | 81（19.2%） |
| T | 967 | 572 | 769 | 768 | 468 |
| C | 701 | 416 | 587 | 580 | 366 |
| MAF(C allele） | 0.420264 | 0.421053 | 0.432891 | 0.430267 | 0.438849 |
| Abbreviations: MAF, minor allele frequency; HC, healthy control; NC, natural clearance; CHB, chronic hepatitis B; LC, liver cirrhosis; HCC, hepatocellular carcinoma; | | | | | |

| Supplementary table 5 : Univariable analysis of predictive factors with regard to the risk of hepatocellular carcinoma among four independent sample case-control studies. | | | | | | | | |
| --- | --- | --- | --- | --- | --- | --- | --- | --- |
| Loci | HC VS. HCC | | NC VS. HCC | | CHB VS. HCC | | LC VS. HCC | |
|  | *P* | OR(95%CI) | *P* | OR(95%CI) | *P* | OR(95%CI) | *P* | OR(95%CI) |
| *MSH2-rs1981928* |  |  |  |  |  |  |  |  |
| TT |  | 1 |  | 1 |  | 1 |  | 1 |
| TA | 0.647 | 0.943(0.732,1.213) | 0.026* | 1.379(1.039,1.829) | 0.849 | 0.975(0.750,1.268) | 0.920 | 0.987(0.758,1.284) |
| AA | 0.699 | 0.927(0.631,1.361) | 0.312 | 0.812(0.542,1.216) | 0.224 | 0.785(0.532,1.159) | 0.162 | 0.758(0.514,1.118) |
| TT/TA+AA | 0.612 | 0.940(0.738,1.196) | 0.158 | 1.210(0.928,1.578) | 0.569 | 0.930(0.724,1.194) | 0.574 | 0.931(0.724,1.196) |
| TT+TA/AA | 0.815 | 0.958(0.670,1.370) | 0.051 | 0.688(0.472,1.002) | 0.217 | 0.796(0.555,1.143) | 0.142 | 0.764(0.533,1.095) |
| T/A | 0.636 | 0.959(0.808,1.139) | 0.994 | 1.001(0.826,1.212) | 0.306 | 0.911(0.763,1.089) | 0.212 | 0.893(0.747,1.067) |
| *MSH2-rs4952887* |  |  |  |  |  |  |  |  |
| CC |  | 1 |  | 1 |  | 1 |  | 1 |
| CT | 0.323 | 0.870(0.659,1.147) | 0.181 | 0.812(0.598,1.102) | 0.045* | 0.748(0.563,0.993) | 0.029* | 0.728(0.548,0.968) |
| TT | 0.069 | 2.518(0.929,6.825) | 0.438 | 0.714(0.305,1.673) | 0.947 | 0.971(0.415,2.271) | 0.467 | 0.740(0.328,1.667) |
| CC/CT+TT | 0.554 | 0.922(0.704,1.207) | 0.143 | 0.802(0.598,1.077) | 0.054 | 0.763(0.579,1.005) | 0.024* | 0.729(0.554,0.960) |
| CC+CT/TT | 0.059 | 2.606(0.964,7.047) | 0.511 | 0.753(0.322,1.757) | 0.915 | 1.047(0.449,2.442) | 0.597 | 0.804(0.358,1.806) |
| C/T | 0.934 | 0.990(0.777,1.261) | 0.133 | 0.819(0.631,1.063) | 0.093 | 0.810(0.634,1.036) | 0.032* | 0.766(0.600,0.977) |
| *MSH2-rs13019654* |  |  |  |  |  |  |  |  |
| GG |  | 1 |  | 1 |  | 1 |  | 1 |
| GT | 0.382 | 0.895(0.698,1.148) | 0.150 | 1.231(0.927,1.633) | 0.918 | 0.986(0.762,1.277) | 0.747 | 0.958(0.739,1.242) |
| TT | 0.517 | 0.841(0.498,1.421) | 0.113 | 0.645(0.375,1.109) | 0.528 | 1.202(0.678,2.131) | 0.658 | 0.884(0.512,1.526) |
| GG/GT+TT | 0.327 | 0.888(0.699,1.126) | 0.478 | 1.101(0.844,1.437) | 0.938 | 1.010(0.788,1.294) | 0.673 | 0.948(0.739,1.215) |
| GG+GT/TT | 0.620 | 0.878(0.524,1.470) | 0.060 | 0.599(0.352,1.022) | 0.510 | 1.209(0.687,2.125) | 0.696 | 0.898(0.525,1.538) |
| G/T | 0.322 | 0.906(0.745,1.102) | 0.817 | 0.975(0.783,1.212) | 0.763 | 1.032(0.840,1.267) | 0.619 | 0.950(0.774,1.165) |
| *MSH2-rs2303428* |  |  |  |  |  |  |  |  |
| TT |  | 1 |  | 1 |  | 1 |  | 1 |
| TC | 0.970 | 0.995(0.777,1.275) | 0.650 | 1.076(0.816,1.149) | 0.872 | 0.979(0.757,1.266) | 0.350 | 1.130(0.874,1.461) |
| CC | 0.887 | 1.031(0.680,1.563) | 0.440 | 0.839(0.537,1.310) | 0.738 | 0.930(0.607,1.424) | 0.533 | 1.148(0.743,1.773 |
| TT/TC+CC | 0.992 | 1.001(0.790,1.269) | 0.852 | 1.025(0.788,1.334) | 0.808 | 0.970(0.759,1.240) | 0.318 | 1.133(0.887,1.449) |
| TT+TC/CC | 0.872 | 1.033(0.695,1.536) | 0.325 | 0.808(0.529,1.234) | 0.764 | 0.940(0.627,1.410) | 0.717 | 1.080(0.713,1.635) |
| T/C | 0.937 | 1.007(0.844,1.202) | 0.747 | 0.968(0.796,1.178) | 0.752 | 0.971(0.809,1.166) | 0.360 | 1.090(0.906,1.311) |
| *MSH2-rs12999145* |  |  |  |  |  |  |  |  |
| AA |  | 1 |  | 1 |  | 1 |  | 1 |
| AG | 0.196 | 0.832(0.629,1.100) | 0.678 | 1.068(0.784,1.455) | 0.547 | 0.915(0.686,1.221) | 0.918 | 0.985(0.739,1.313) |
| GG | 0.871 | 0.973(0.694,1.363) | 0.617 | 0.911(0.633,1.311) | 0.816 | 1.042(0.736,1.477) | 0.456 | 1.142(0.806,1.618) |
| AA/AG+GG | 0.306 | 0.871(0.668,1.135) | 0.928 | 1.014(0.758,1.355) | 0.720 | 0.951(0.724,1.250) | 0.836 | 1.029(0.784,1.350) |
| AA+AG/GG | 0.511 | 1.099(0.829,1.458) | 0.389 | 0.874(0.644,1.187) | 0.507 | 1.105(0.824,1.481) | 0.345 | 1.153(0.858,1.549) |
| A/G | 0.802 | 0.979(0.830,1.155) | 0.644 | 0.958(0.797,1.151) | 0.872 | 1.014(0.854,1.204) | 0.498 | 1.061(0.893,1.261) |
| *MSH3-rs1428030* |  |  |  |  |  |  |  |  |
| TT |  | 1 |  | 1 |  | 1 |  | 1 |
| TC | 0.629 | 0.939(0.728,1.211) | 0.629 | 1.073(0.807,1.427) | 0.312 | 0.873(0.671,1.136) | 0.355 | 0.883(0.678,1.150) |
| CC | 0.213 | 1.251(0.879,1.781) | 0.619 | 1.103(0.750,1.621) | 0.136 | 1.329(0.914,1.931) | 0.190 | 1.283(0.884,1.862) |
| TT/TC+CC | 0.941 | 1.009(0.796,1.280) | 0.565 | 1.081(0.830,1.408) | 0.775 | 0.965(0.753,1.235) | 0.786 | 0.966(0.754,1.238) |
| TT+TC/CC | 0.126 | 1.292(0.930,1.794) | 0.730 | 1.065(0.744,1.525) | 0.046* | 1.426(1.007,2.020) | 0.076 | 1.369(0.968,1.936) |
| T/C | 0.402 | 1.076(0.906,1.278) | 0.547 | 1.061(0.875,1.285) | 0.422 | 1.076(0.900,1.286) | 0.480 | 1.067(0.892,1.276) |
| *MSH3-rs2112416* |  |  |  |  |  |  |  |  |
| TT |  | 1 |  | 1 |  | 1 |  | 1 |
| TA | 0.067 | 0.787(0.609,1.017) | 0.070 | 0.768(0.577,1.022) | 0.021 | 0.730(0.559,0.953) | 0.028 | 0.741(0.568,0.968) |
| AA | 0.125 | 1.314(0.927,1.863) | 0.694 | 1.080(0.735,1.586) | 0.256 | 1.235(0.858,1.778) | 0.282 | 1.221(0.849,1.756) |
| TT/TA+AA | 0.372 | 0.897(0.707,1.139) | 0.206 | 0.842(0.645,1.099) | 0.154 | 0.835(0.651,1.070) | 0.177 | 0.843(0.657,1.080) |
| TT+TA/AA | 0.016 | 1.487(1.076,2.056) | 0.235 | 1.239(0.869,1.766) | 0.029 | 1.457(1.040,2.041) | 0.038 | 1.428(1.020,2.000) |
| T/A | 0.000002 | 1.501(1.269,1.777) | 0.789 | 0.974(0.806,1.179) | 0.908 | 1.011(0.846,1.207) | 0.908 | 1.011(0.846,1.207) |
| *MSH3-rs26279* |  |  |  |  |  |  |  |  |
| GG |  | 1 |  | 1 |  | 1 |  | 1 |
| GA | 0.561 | 1.076(0.841,1.377) | 0.843 | 1.028(0.782,1.352) | 0.799 | 0.967(0.749,1.249) | 0.424 | 0.901(0.698,1.163) |
| AA | 0.391 | 1.273(0.733,2.212) | 0.825 | 0.936(0.522,1.679) | 0.307 | 0.757(0.444,1.291) | 0.848 | 0.947(0.544,1.649) |
| GG/GA+AA | 0.446 | 1.097(0.865,1.391) | 0.907 | 1.016(0.781,1.321) | 0.594 | 0.935(0.732,1.196) | 0.435 | 0.907(0.709,1.159) |
| GG+GA/AA | 0.442 | 1.238(0.718,2.132) | 0.794 | 0.926(0.522,1.645) | 0.321 | 0.767(0.455,1.295) | 0.966 | 0.988(0.573,1.704) |
| G/A | 0.367 | 1.094(0.900,1.331) | 0.998 | 1.000(0.806,1.240) | 0.410 | 0.920(0.753,1.123) | 0.515 | 0.936(0.766,1.143) |
| *MSH3-rs26779* |  |  |  |  |  |  |  |  |
| CC |  | 1 |  | 1 |  | 1 |  | 1 |
| CT | 0.218 | 0.851(0.659,1.100) | 0.175 | 0.822(0.619,1.091) | 0.018* | 0.724(0.554,0.946) | 0.081 | 0.789(0.604,1.029) |
| TT | 0.205 | 1.254(0.884,1.779) | 0.259 | 1.255(0.846,1.862) | 0.913 | 1.020(0.711,1.464) | 0.361 | 1.186(0.832,1.708) |
| CC/CT+TT | 0.613 | 0.940(0.741,1.194) | 0.511 | 0.915(0.702,1.193) | 0.067 | 0.792(0.617,1.016) | 0.292 | 0.875(0.682,1.122) |
| CC+CT/TT | 0.062 | 1.362(0.984,1.885) | 0.079 | 1.389(0.963,2.004) | 0.257 | 1.212(0.869,1.690) | 0.088 | 1.342(0.957,1.883) |
| C/T | 0.550 | 1.054(0.887,1.252) | 0.657 | 1.044(0.863,1.264) | 0.482 | 0.938(0.786,1.121) | 0.897 | 1.012(0.847,1.210) |
| *MSH3-rs33002* |  |  |  |  |  |  |  |  |
| AA |  | 1 |  | 1 |  | 1 |  | 1 |
| AT | 0.027* | 0.749(0.579,0.967) | 0.986 | 0.997(0.752,1.323) | 0.535 | 0.920(0.706,1.198) | 0.680 | 0.946(0.726,1.232) |
| TT | 0.677 | 0.927(0.647,1.327) | 0.772 | 1.060(0.714,1.574) | 0.403 | 1.174(0.806,1.711) | 0.621 | 1.099(0.757,1.595) |
| AA/AT+TT | 0.052 | 0.787(0.618,1.002) | 0.926 | 1.013(0.776,1.321) | 0.831 | 0.973(0.759,1.249) | 0.876 | 0.980(0.764,1.258) |
| AA+AT/TT | 0.599 | 1.092(0.786,1.518) | 0.748 | 1.062(0.737,1.529) | 0.247 | 1.229(0.867,1.741) | 0.480 | 1.132(0.802,1.598) |
| A/T | 0.280 | 0.962(0.810,1.142) | 0.816 | 1.023(0.846,1.237) | 0.666 | 1.040(0.871,1.242) | 0.803 | 1.023(0.856,1.222) |
| *MSH3-rs33008* |  |  |  |  |  |  |  |  |
| GG |  | 1 |  | 1 |  | 1 |  | 1 |
| GC | 0.008* | 0.712(0.555,0.914) | 0.840 | 1.030(0.776,1.365) | 0.610 | 1.071(0.823,1.394) | 0.166 | 0.832(0.641,1.079) |
| CC | 0.743 | 0.921(0.563,1.506) | 0.353 | 0.783(0.467,1.313) | 0.637 | 0.888(0.542,1.455) | 0.817 | 0.942(0.567,1.564) |
| GG/GC+CC | 0.013* | 0.739(0.582,0.937) | 0.883 | 0.980(0.752,1.278) | 0.776 | 1.037(0.809,1.329) | 0.191 | 0.848(0.662,1.086) |
| GG+GC/CC | 0.824 | 1.056(0.652,1.710) | 0.324 | 0.775(0.467,1.286) | 0.562 | 0.866(0.534,1.407) | 0.966 | 1.011(0.615,1.662) |
| G/C | 0.054 | 0.827（0.682,1.004） | 0.584 | 0.941(0.758,1.169) | 0.738 | 0.998(0.815,1.224) | 0.296 | 0.898(0.734,1.099) |
| *MSH3-rs12513549* |  |  |  |  |  |  |  |  |
| GG |  | 1 |  | 1 |  | 1 |  | 1 |
| GT | 0.699 | 1.050(0.820,1.345) | 0.530 | 0.916(0.696,1.205) | 0.403 | 0.896(0.693,1.159) | 0.328 | 0.880(0.681,1.137) |
| TT | 0.732 | 1.080(0.695,1.678) | 0.706 | 1.101(0.668,1.814) | 0.198 | 0.750(0.483,1.163) | 0.898 | 0.971(0.615,1.532) |
| GG/GT+TT | 0.654 | 1.055(0.834,1.335) | 0.664 | 0.944(0.727,1.225) | 0.248 | 0.866(0.679,1.105) | 0.370 | 0.894(0.701,1.142) |
| GG+GT/TT | 0.797 | 1.058(0.690,1.623) | 0.586 | 1.144(0.705,1.858) | 0.269 | 0.787(0.515,1.203) | 0.904 | 1.027(0.660,1.599) |
| G/T | 0.642 | 1.045(0.868,1.258) | 0.911 | 0.988(0.805,1.214) | 0.162 | 0.874(0.723,1.056) | 0.517 | 0.939(0.776,1.136) |
| *MSH3-rs1805355* |  |  |  |  |  |  |  |  |
| GG |  | 1 |  | 1 |  | 1 |  | 1 |
| GA | 0.597 | 0.933(0.723,1.205) | 0.691 | 1.059(0.798,1.406) | 0.505 | 0.914(0.701,1.191) | 0.459 | 0.904(0.693,1.180) |
| AA | 0.289 | 1.214(0.848,1.738) | 0.297 | 1.235(0.830,1.839) | 0.095 | 1.384(0.945,2.026) | 0.228 | 1.262(0.864,1.841) |
| GG/GA+AA | 0.965 | 0.995(0.783,1.264) | 0.477 | 1.101(0.844,1.436) | 0.970 | 1.005(0.783,1.289) | 0.863 | 0.978(0.762,1.256) |
| GG+GA/AA | 0.175 | 1.259(0.902,1.757) | 0.335 | 1.200(0.828,1.738) | 0.040* | 1.451(1.017,2.070) | 0.112 | 1.330(0.936,1.891) |
| G/A | 0.511 | 1.060(0.892,1.259) | 0.309 | 1.105(0.911,1.340) | 0.294 | 1.101(0.920,1.318) | 0.497 | 1.064(0.889,1.274) |
| *MSH3-rs181747* |  |  |  |  |  |  |  |  |
| TT |  | 1 |  | 1 |  | 1 |  | 1 |
| TC | 0.296 | 0.871(0.671,1.129) | 0.414 | 0.887(0.664,1.183) | 0.114 | 0.805(0.614,1.054) | 0.149 | 0.820(0.626,1.074) |
| CC | 0.068 | 1.373(0.977,1.928) | 0.310 | 1.215(0.835,1.767) | 0.145 | 1.302(0.913,1.857) | 0.035* | 1.475(1.029,2.116) |
| TT/TC+CC | 0.907 | 0.986(0.774,1.255) | 0.826 | 0.970(0.742,1.269) | 0.496 | 0.916(0.713,1.178) | 0.723 | 0.955(0.743,1.229) |
| TT+TC/CC | 0.013* | 1.479(1.084,2.017) | 0.138 | 1.295(0.920,1.823) | 0.020* | 1.467(1.062,2.026) | 0.003* | 1.644(1.182,2.287) |
| T/C | 0.216 | 1.113(0.939,1.319) | 0.507 | 1.066(0.883,1.287) | 0.442 | 1.071(0.899,1.278) | 0.191 | 1.125(0.943,1.343) |
| *MSH3-rs32950* |  |  |  |  |  |  |  |  |
| AA |  | 1 |  | 1 |  | 1 |  | 1 |
| AG | 0.961 | 0.994(0.768,1.285) | 0.216 | 0.834(0.625,1.112) | 0.101 | 0.798(0.609,1.045) | 0.437 | 0.899(0.687,1.176) |
| GG | 0.037* | 1.469(1.024,2.107) | 0.707 | 1.079(0.726,1.605) | 0.655 | 0.921(0.640,1.323) | 0.246 | 1.248(0.859,1.814) |
| AA/AG+GG | 0.470 | 1.093(0.859,1.390) | 0.399 | 0.890(0.680,1.166) | 0.144 | 0.829(0.644,1.066) | 0.846 | 0.975(0.758,1.254) |
| AA+AG/GG | 0.024* | 1.474(1.053,2.063) | 0.357 | 1.188(0.823,1.715) | 0.824 | 1.039(0.744,1.450) | 0.118 | 1.319(0.932,1.867) |
| A/G | 0.095 | 1.160(0.975,1.381) | 0.906 | 0.988(0.815,1.199) | 0.361 | 0.920(0.769,1.100) | 0.506 | 1.063(0.887,1.275) |
| *MSH3-rs863221* |  |  |  |  |  |  |  |  |
| TT |  | 1 |  | 1 |  | 1 |  | 1 |
| GT | 0.546 | 0.925(0.717,1.193) | 0.180 | 0.823(0.620,1.094) | 0.029* | 0.744(0.570,0.969) | 0.131 | 0.815(0.625,1.063) |
| GG | 0.091 | 1.365(0.952,1.958) | 0.541 | 1.133(0.759,1.693) | 0.796 | 1.050(0.725,1.521) | 0.329 | 1.206(0.828,1.756) |
| TT/GT+GG | 0.890 | 1.017(0.802,1.289) | 0.398 | 0.892(0.684,1.163) | 0.095 | 0.810(0.633,1.037) | 0.389 | 0.897(0.700,1.149) |
| TT+GT/GG | 0.044* | 1.418(1.010,1.991) | 0.247 | 1.249(0.857,1.818) | 0.257 | 1.221(0.864,1.726) | 0.108 | 1.335(0.939,1.899) |
| T/G | 0.265 | 1.104(0.927,1.315) | 0.983 | 0.998(0.822,1.211) | 0.530 | 0.944(0.789,1.130) | 0.854 | 1.017(0.849,1.219) |
| *MSH3-rs3776968* |  |  |  |  |  |  |  |  |
| CC |  | 1 |  | 1 |  | 1 |  | 1 |
| CT | 0.002* | 0.673(0.525,0.862) | 0.488 | 1.104(0.834,1.463) | 0.637 | 1.065(0.821,1.381) | 0.068 | 0.787(0.608,1.018) |
| TT | 0.510 | 0.855(0.535,1.364) | 0.398 | 0.808(0.492,1.325) | 0.867 | 0.960(0.596,1.547) | 0.699 | 0.909(0.559,1.476) |
| CC/CT+TT | 0.003* | 0.698(0.551,0.884) | 0.765 | 1.041(0.800,1.355) | 0.722 | 1.046(0.818,1.337) | 0.083 | 0.805(0.630,1.028) |
| CC+CT/TT | 0.947 | 1.016(0.644,1.603) | 0.310 | 0.778(0.480,1.262) | 0.786 | 0.937(0.588,1.495) | 0.986 | 1.004(0.625,1.612) |
| C/T | 0.020* | 0.800(0.663,0.966) | 0.832 | 0.977(0.790,1.208) | 0.864 | 1.017(0.834,1.241) | 0.169 | 0.871(0.716,1.060) |
| *MSH3-rs40139* |  |  |  |  |  |  |  |  |
| AA |  | 1 |  | 1 |  |  |  | 1 |
| AG | 0.909 | 1.015(0.787,1.309) | 0.159 | 0.815(0.614,1.083) | 0.187 | 0.836(0.641,1.091) | 0.447 | 0.902(0.693,1.176) |
| GG | 0.104 | 1.344(0.941,1.920) | 0.667 | 1.091(0.733,1.625) | 0.695 | 0.930(0.648,1.335) | 0.276 | 1.229(0.848,1.783) |
| AA/AG+GG | 0.489 | 1.088(0.857,1.380) | 0.330 | 0.876(0.671,1.144) | 0.234 | 0.860(0.671,1.013) | 0.828 | 0.973(0.759,1.247) |
| AA+AG/GG | 0.089 | 1.334(0.957,1.860) | 0.296 | 1.217(0.842,1.759) | 0.894 | 1.023(0.733,1.427) | 0.140 | 1.297(0.918,1.832) |
| A/G | 0.171 | 1.128(0.949,1.341) | 0.877 | 0.985(0.814,1.192) | 0.439 | 0.932(0.781,1.113) | 0.551 | 1.056(0.883,1.263) |
| *MSH6-rs1042821* |  |  |  |  |  |  |  |  |
| GG |  | 1 |  | 1 |  | 1 |  | 1 |
| GA | 0.013* | 0.727(0.565,0.936) | 0.619 | 0.931(0.704,1.233) | 0.494 | 0.913(0.702,1.186) | 0.683 | 1.057(0.810,1.379) |
| AA | 0.279 | 1.348(0.785,2.317) | 0.030* | 2.110(1.074,4.143) | 0.045* | 1.834(1.013,3.320) | 0.422 | 1.249(0.726,2.148) |
| GG/GA+AA | 0.048* | 0.784(0.616,0.997) | 0.880 | 1.021(0.780,1.336) | 0.942 | 0.991(0.771,1.273) | 0.535 | 1.083(0.841,1.395) |
| GG+GA/AA | 0.123 | 1.521(0.892,2.593) | 0.024* | 2.163(1.109,4.217) | 0.033* | 1.894(1.054,3.404) | 0.455 | 1.226(0.718,2.093) |
| G/A | 0.276 | 0.895(0.734,1.092) | 0.347 | 1.115(0.889,1.397) | 0.485 | 1.078(0.874,1.329) | 0.418 | 1.091(0.884,1.347) |
| *MSH6-rs2348244* |  |  |  |  |  |  |  |  |
| TT |  | 1 |  | 1 |  | 1 |  | 1 |
| TC | 0.341 | 1.138(0.872,1.486) | 0.409 | 1.132(0.843,1.521) | 0.875 | 1.023(0.775,1.350) | 0.858 | 1.026(0.777,1.354) |
| CC | 0.448 | 1.140(0.813,1.600) | 0.512 | 1.134(0.779,1.650) | 0.786 | 1.050(0.738,1.493) | 0.688 | 1.075(0.755,1.531) |
| TT/TC+CC | 0.310 | 1.139(0.886,1.464) | 0.378 | 1.133(0.858,1.495) | 0.824 | 1.030(0.793,1.338) | 0.773 | 1.039(0.800,1.350) |
| TT+TC/CC | 0.721 | 1.056(0.783,1.424) | 0.759 | 1.053(0.756,1.468) | 0.824 | 1.036(0.760,1.412) | 0.719 | 1.059(0.776,1.445) |
| T/C | 0.376 | 1.079(0.912,1.276) | 0.444 | 1.075(0.893,1.295) | 0.785 | 1.025(0.861,1.219) | 0.694 | 1.036(0.870,1.233) |
| Abbreviations: HC, healthy control; NC, natural clearance; CHB, chronic hepatitis B; LC, liver cirrhosis; HCC, hepatocellular carcinoma; * : *P*<0.05. | | | | | | | | |

| Supplementary table 6 : Haplotype analysis for 7 SNPs near *MSH2*, *MSH6* gene in HC vs. HCC, NC vs. HCC, CHB vs. HCC, and LC vs HCC groups by Haploview 4.2. | | | | | |
| --- | --- | --- | --- | --- | --- |
| Group | Haplotype | Freq. | Case, Control Ratios | *P* value | OR(95%CI) |
| HC vs. HCC |  |  |  |  |  |
|  | Block 1 |  |  |  |  |
|  | AG | 0.518 | 438.3 : 403. 7, 867.4 : 812.6 | 0.8410 | 1.015(0.860,1.198) |
|  | GG | 0.240 | 210.2 : 631.8, 395.6 : 1284.4 | 0.4331 | 1.077(0.889,1.306) |
|  | GT | 0.238 | 189.8 : 652.2, 411.4 : 1268.6 | 0.2799 | 0.900(0.739,1.095) |
| NC vs. HCC |  |  |  |  |  |
|  | Block 1 |  |  |  |  |
|  | AG | 0.517 | 438.6 : 403.4, 509.5 : 482.5 | 0.7588 | 1.032(0.858,1.240) |
|  | GG | 0.250 | 210.0 : 632.0, 248.5 : 743.5 | 0.9549 | 0.993(0.803,1.227) |
|  | GT | 0.231 | 190.0 : 652.0, 233.5 : 758.5 | 0.6240 | 0.945(0.760,1.176) |
| CHB vs. HCC |  |  |  |  |  |
|  | Block 1 |  |  |  |  |
|  | AG | 0.525 | 438.4 : 403.6, 722.2 : 647.8 | 0.7638 | 0.973(0.819,1.155) |
|  | GG | 0.249 | 210.2 : 631.8, 339.9 : 1030. 1 | 0.9351 | 1.007(0.825,1.228) |
|  | GT | 0.224 | 189.8: 652.2, 304.7 : 1065.3 | 0.8704 | 1.018(0.828,1.250) |
|  | Block 2 |  |  |  |  |
|  | GC | 0.432 | 366.8 : 473.2, 587.3 : 782.7 | 0.7130 | 1.035(0.870,1.231) |
|  | GT | 0.355 | 288.1 : 551.9, 496.7 : 873.3 | 0.3501 | 0.916(0.765,1.097) |
|  | AT | 0.210 | 183.1: 656.9, 280.2 : 1089. 8 | 0.4491 | 1.084(0.879,1.338) |
| LC vs. HCC |  |  |  |  |  |
|  | Block 1 |  |  |  |  |
|  | AG | 0.531 | 438.2 : 403.8, 729.0 : 629. 0 | 0.4548 | 0.935(0.787,1.111) |
|  | GT | 0.233 | 189.8 : 652.2, 322.7 : 1035.3 | 0.5092 | 0.934(0.761,1.145) |
|  | GG | 0.232 | 210.2 : 631.8, 300.9 : 1057.1 | 0.1295 | 1.167(0.954,1.428) |
|  | Block 2 |  |  |  |  |
|  | GC | 0.429 | 366.4 : 473.6, 576.4 : 781.6 | 0.5896 | 1.048(0.881,1.247) |
|  | GT | 0.359 | 288.5 : 551.5, 501.4 : 856.6 | 0.2212 | 0.896(0.748,1.072) |
|  | AT | 0.207 | 182.8 : 657.2, 272.1 : 1085.9 | 0.3338 | 1.112(0.901,1.373) |
| Abbreviations: HC, healthy control; NC, natural clearance; CHB, chronic hepatitis B; LC, liver cirrhosis; HCC, hepatocellular carcinoma; SNPs, single nucleotide polymorphisms; *:*P*<0.05.  HC vs. HCC:Two SNPs of Block 1, including rs12999145_G, rs13019654_T, were in LD.  NC vs. HCC:Two SNPs of Block 1, including rs12999145_G, rs13019654_T, were in LD.  CHB vs. HCC:Two SNPs of Block 1, including rs12999145_G, rs13019654_T, were in LD; Two SNPs of Block 2, including rs1042821_A, rs2348244_C, were in LD.  LC vs. HCC:Two SNPs of Block 1, including rs12999145_G, rs13019654_T, were in LD; Two SNPs of Block 2, including rs1042821_A, rs2348244_C, were in LD. | | | | | |

| Supplementary table 7 :The negative results of additive interaction analysis of SNPs between HC and HCC group. | | | | | | | |
| --- | --- | --- | --- | --- | --- | --- | --- |
| SNP1 | SNP2 | HC | HCC | B | *P* | OR(95%CI) | RERI/AP/S |
| rs4952887 | rs12999145 |  |  |  |  |  |  |
| CC+CT | AG+GG | 631 | 303 |  |  | 1 |  |
| CC+CT | AA | 200 | 107 | 0.217 | 0.317 | 1.242(0.812,1.901) | RERI:1.362(-1.234.3.957) |
| TT | AG+GG | 0 | 0 | 0 | 0 | 1 | AP:0.523(0.039,1.007) |
| TT | AA | 7 | 9 | 0.957 | 0.150 | 2.605(0.708,9.579) | S:6.618(0.848,51.640) |
| rs4952887 | rs3776968 |  |  |  |  |  |  |
| CC+CT | CT+TT | 431 | 176 |  |  | 1 |  |
| CC+CT | CC | 396 | 232 | 0.472 | 0.133 | 1.603(0.866.2.969) | RERI:6.084(-3.193,15.361) |
| TT | CT+TT | 3 | 3 | -0.562 | 0.581 | 0.570(0.077,4.194) | AP:0.838(0.594,1.082) |
| TT | CC | 4 | 6 | 1.982 | **0.021** | 7.260(1.346,39.155) | S:36.114(0.088,14785.550) |
| rs1981928 | rs3776968 |  |  |  |  |  |  |
| TA+AA | CT+TT | 377 | 158 |  |  | 1 |  |
| TA+AA | CC | 349 | 209 | 0.429 | 0.181 | 1.536(0.819,2.880) | RERI:0.263(-0.558,1.084) |
| TT | CT+TT | 56 | 22 | -0.373 | 0.284 | 0.689(0.348,1.362) | AP:0.177(-0.317,0.670) |
| TT | CC | 49 | 29 | 0.397 | 0.353 | 1.487(0.643,3.441) | S:2.172(0.145,32.467) |
| rs1981928 | rs863221 |  |  |  |  |  |  |
| TA+AA | TT+GT | 637 | 308 |  |  | 1 |  |
| TA+AA | GG | 84 | 56 | 0.732 | 0.093 | 2.079(0.885,4.883) | RERI:0.904(-1.570,3.378) |
| TT | TT+GT | 93 | 42 | -0.293 | 0.296 | 0.746(0.431,1.293) | AP:0.331(-0.311,0.974) |
| TT | GG | 12 | 9 | 1.004 | 0.120 | 2.730(0.771,9.668) | S:2.095(0.398,11.035) |
| rs1981928 | rs33008 |  |  |  |  |  |  |
| TA+AA | GC+CC | 339 | 147 |  |  | 1 |  |
| TA+AA | GG | 384 | 220 | -0.514 | 0.107 | 0.598(0.320,1.117) | RERI:0.441(-0.004,0.885) |
| TT | GC+CC | 56 | 21 | -0.502 | 0.155 | 0.605(0.303,1.210) | AP:0.684(0.087,1.281) |
| TT | GG | 49 | 30 | -0.440 | 0.311 | 0.644(0.275,1.508) | S:0.447(0.180,1.111) |
| rs12999145 | rs1042821 |  |  |  |  |  |  |
| AG+GG | GA+AA | 288 | 119 |  |  | 1 |  |
| AG+GG | GG | 332 | 183 | 0.232 | 0.183 | 0.261(0.897,1.774) | RERI:-0.329(-1.113,0.456) |
| AA | GA+AA | 73 | 40 | 0.388 | 0.192 | 1.474(0.823,2.640) | AP:-0.234(-0.813,0.345) |
| AA | GG | 129 | 76 | 0.341 | 0.203 | 1.407(0.832,2.378) | S:0.553(0.152,2.007) |
| rs12999145 | rs2348244 |  |  |  |  |  |  |
| AG+GG | TT | 223 | 102 |  |  | 1 |  |
| AG+GG | TC+CC | 402 | 200 | 0.169 | 0.351 | 1.184(0.831,1.687) | RERI:0.184(-0.520,0.887) |
| AA | TT | 65 | 30 | 0.138 | 0.679 | 1.149(0.597,2.211) | AP:0.121(-0.333,0.576 |
| AA | TC+CC | 144 | 85 | 0.416 | 0.114 | 1.516(0.905,2.540) | S:1.553(0.202,11.963) |
| rs12999145 | rs3776968 |  |  |  |  |  |  |
| AG+GG | CT+TT | 342 | 135 |  |  | 1 |  |
| AG+GG | CC | 285 | 168 | 0.495 | 0.122 | 1.640(0.876,3.070) | RERI:0.048(-0.787,0.883) |
| AA | CT+TT | 94 | 46 | 0.257 | 0.373 | 1.292(0.735,2.272) | AP:0.024(-0.393,0.442) |
| AA | CC | 115 | 70 | 0.684 | 0.080 | 1.981(0.923,4.255) | S:1.052(0.437,2.530) |
| rs12999145 | rs863221 |  |  |  |  |  |  |
| AG+GG | TT+GT | 560 | 257 |  |  | 1 |  |
| AG+GG | GG | 62 | 44 | 0.725 | 0.092 | 2.121(0.885,5.082) | RERI:0.584(-1.762,2.930) |
| AA | TT+GT | 175 | 94 | 0.198 | **0.0382** | 1.219(0.782,1.902) | AP:0.200(-0.535,0.934) |
| AA | GG | 34 | 21 | 1.073 | 0.052 | 2.924(0.991,8.626) | S:1.436(0.309,6.667) |
| rs12999145 | rs33008 |  |  |  |  |  |  |
| AG+GG | GC+CC | 305 | 126 |  |  | 1 |  |
| AG+GG | GG | 319 | 177 | -0.456 | 0.154 | 0.634(0.339,1.186) | RERI:0.056(-0.477,0.588) |
| AA | GC+CC | 93 | 43 | 0.134 | 0.641 | 1.143(0.645,2.026) | AP:0.067(-0.569,0.702) |
| AA | GG | 116 | 73 | -0.183 | 0.635 | 0.833(0.392,1.771) | S:0.751(0.061,9.219) |
| rs1042821 | rs3776968 |  |  |  |  |  |  |
| GA+AA | CT+TT | 191 | 72 |  |  | 1 |  |
| GA+AA | CC | 168 | 87 | 0.553 | 0.121 | 1.738(0.864,3.497) | RERI:-0.060(-0.786,0.667) |
| GG | CT+TT | 240 | 109 | 0.228 | 0.294 | 1.256(0.820,1.924) | AP:-0.031(-0.407,0.345) |
| GG | CC | 219 | 149 | 0.660 | 0.058 | 1.934(0.978,3.824) | S:0.940(0.452,1.955) |
| rs1042821 | rs863221 |  |  |  |  |  |  |
| GA+AA | TT+GT | 326 | 134 |  |  | 1 |  |
| GA+AA | GG | 30 | 22 | 1.339 | **0.008** | 3.817(1.414,10.301) | RERI:-2.046(-4.382,0.290) |
| GG | TT+GT | 393 | 215 | 0.281 | 0.087 | 1.325(0.960,1.828) | AP:-0.977(-2.242,0.288) |
| GG | GG | 64 | 42 | 0.739 | 0.111 | 2.094(0.843,5.199) | S:0.348(0.128,0.947) |
| rs1042821 | rs33008 |  |  |  |  |  |  |
| GA+AA | GC+CC | 169 | 66 |  |  | 1 |  |
| GA+AA | GG | 189 | 93 | 0.391 | 0.272 | 0.677(0.377,1.359) | RERI:-0.116(-0.600,0.367) |
| GG | GC+CC | 224 | 103 | 0.196 | 0.381 | 1.216(0.785,1.884) | AP:-0.150(-0.757,0.457) |
| GG | GG | 233 | 155 | -0.253 | 0.476 | 0.777(0.388,1.556) | S:2.087(0.013,347.857) |
| rs2348244 | rs863221 |  |  |  |  |  |  |
| TT | TT+GT | 255 | 116 |  |  | 1 |  |
| TT | GG | 33 | 14 | 0.772 | 0.170 | 2.059(0.733,5.782) | RERI:0.345(-1.346,2.036) |
| TC+CC | TT+GT | 476 | 233 | 0.183 | 0.285 | 1.201(0.859,1.678) | AP:0.127(-0.474,0.728) |
| TC+CC | GG | 63 | 51 | 0.997 | 0.034 | 2.709(1.077,6.813) | S:1.253(0.389,4.038) |
| rs2348244 | rs33008 |  |  |  |  |  |  |
| TT | GC+CC | 143 | 60 |  |  | 1 |  |
| TT | GG | 145 | 72 | -0.789 | **0.031** | 0.454(0.222,0.932) | RERI:0.379(0.049,0.708) |
| TC+CC | GC+CC | 255 | 107 | -0.121 | 0.597 | 0.886(0.566,1.387) | AP:0.527(-0.014,1.068) |
| TC+CC | GG | 286 | 178 | -0.330 | 0.351 | 0.719(0.360,1.437) | S:0.426(0.230,0.788) |
| rs3776968 | rs863221 |  |  |  |  |  |  |
| CT+TT | TT+GT | 428 | 173 |  |  | 1 |  |
| CT+TT | GG | 6 | 6 | 1.187 | 0.112 | 3.276(0.759,14.133) | RERI:-0.917(-4.789,2.955) |
| CC | TT+GT | 307 | 176 | 0.555 | 0.096 | 1.741(0.906,3.346) | AP:-0.296(-1.570,0.978) |
| CC | GG | 90 | 59 | 1.132 | 0.063 | 3.103(0.940,10.247) | S:0.696(0.185,2.618) |
| rs3776968 | rs33008 |  |  |  |  |  |  |
| CT+TT | GC+CC | 385 | 163 |  |  | 1 |  |
| CT+TT | GG | 46 | 18 | -0.424 | 0.254 | 0.654(0.316,1.355) | RERI:-0.218(-1.851,1.414) |
| CC | GC+CC | 13 | 6 | 0.484 | 0.387 | 1.622(0.542,4.855) | AP:-0.206(-1.748,1.335) |
| CC | GG | 387 | 231 | 0.057 | 0.787 | 1.056(0.701,1.597) | S:0.212(0.000,183.884) |
| rs863221 | rs33008 |  |  |  |  |  |  |
| TT+TG | GC+CC | 394 | 165 |  |  | 1 |  |
| TT+TG | GG | 338 | 185 | 0.415 | 0.205 | 0.661(0.348,1.253) | RERI:-0.647(-4.798,3.504) |
| GG | GC+CC | 4 | 2 | 0.885 | 0.413 | 2.422(0.291,20.167) | AP:-0.451(-3.355,2.454) |
| GG | GG | 92 | 63 | 0.362 | 0.434 | 1.436(0.580,3.555) | S:0.403(0.008,20.679) |
| rs2303428 | rs2348244 |  |  |  |  |  |  |
| TT | TT | 120 | 53 |  |  |  |  |
| TT | TC+CC | 242 | 128 | 0.423 | 0.081 | 1.526(0.949,2.456) | RERI:-0.484(-1.197,0.229) |
| TC+CC | TT | 168 | 79 | 0.205 | 0.460 | 1.228(0.712,2.118) | AP:-0.381(-0.903,0.141) |
| TC+CC | TC+CC | 302 | 155 | 0.239 | 0.354 | 1.270(0.767,2.103) | S:0.358(0.112,1.143) |
| rs2303428 | rs863221 |  |  |  |  |  |  |
| TT | TT+GT | 319 | 154 |  |  |  |  |
| TT | GG | 42 | 26 | 0.823 | 0.095 | 2.278(0.867,5.987) | RERI:-0.208(-1.624,1.209) |
| TC+CC | TT+GT | 416 | 194 | -0.049 | 0.796 | 0.952(0.657,1.380) | AP:-0.103(-0.826,0.621) |
| TC+CC | GG | 54 | 39 | 0.704 | 0.139 | 2.022(0.796,5.134) | S:0.831(0.243,2.841) |
| rs2303428 | rs33008 |  |  |  |  |  |  |
| TT | GC+CC | 167 | 71 |  |  |  |  |
| TT | GG | 197 | 111 | -0.61 | 0.083 | 0.543(0.273,1.083) | RERI:0.264(-0.065,0.594) |
| TC+CC | GC+CC | 231 | 97 | -0.247 | 0.321 | 0.781(0.480,1.272) | AP:0.449(-0.170,1.068) |
| TC+CC | GG | 236 | 137 | -0.53 | 0.144 | 0.588(0.289,1.198) | S:0.609(0.378,0.982) |
| rs2303428 | rs32950 |  |  |  |  |  |  |
| TT | AA | 46 | 29 |  |  |  |  |
| TT | AG+GG | 313 | 149 | 0.446 | 0.330 | 1.562(0.636,3.834) | RERI:-0.092(-0.861,0.677) |
| TC+CC | AA | 52 | 38 | -0.005 | 0.991 | 0.995(0.456,2.173) | AP:-0.063(-0.573,0.447) |
| TC+CC | AG+GG | 420 | 190 | 0.382 | 0.423 | 1.465(0.576,3.722) | S:0.835(0.234,2.979) |
| rs32950 | rs4952887 |  |  |  |  |  |  |
| AA | CC+CT | 96 | 66 |  |  |  |  |
| AA | TT | 2 | 1 | 0.65 | 0.639 | 1.915(0.126,29.000) | RERI:0.821(-5.044,6.687) |
| AG+GG | CC+CT | 726 | 330 | 0.417 | 0.335 | 1.518(0.650,3.546) | AP:0.252(-1.393,1.898) |
| AG+GG | TT | 5 | 8 | 1.18 | 0.150 | 3.256(0.651,16.272) | S:1.573(0.043,57.955) |
| rs32950 | rs1981928 |  |  |  |  |  |  |
| AA | TA+AA | 86 | 57 |  |  |  |  |
| AA | TT | 12 | 10 | 0.349 | 0.512 | 1.418(0.499,4.030) | RERI:-0.848(-2.275,0.580) |
| AG+GG | TA+AA | 633 | 298 | 0.478 | 0.271 | 1.613(0.689,3.776) | AP:-0.716(-1.952,0.519) |
| AG+GG | TT | 93 | 41 | 0.168 | 0.734 | 1.183(0.449,3.116) | S:0.178(0.010,3.216) |
| rs32950 | rs12999145 |  |  |  |  |  |  |
| AA | AG+GG | 61 | 43 |  |  |  |  |
| AA | AA | 37 | 24 | 0.304 | 0.450 | 1.355(0.615,2.985) | RERI:-0.008(-0.971,0.956) |
| AG+GG | AG+GG | 565 | 250 | 0.462 | 0.311 | 1.587(0.649,3.879) | AP:-0.004(-0.502,0.494) |
| AG+GG | AA | 168 | 90 | 0.66 | 0.189 | 1.934(0.723,5.178) | S:0.992(0.357,2.756) |
| rs32950 | rs2348244 |  |  |  |  |  |  |
| AA | TT | 32 | 14 |  |  |  |  |
| AA | TC+CC | 66 | 53 | 0.424 | 0.313 | 1.528(0.670,3.482) | RERI:-0.219(-1.295,0.858) |
| AG+GG | TT | 254 | 112 | 0.592 | 0.251 | 1.807(0.658,4.967) | AP:-0.103(-0.573,0.367) |
| AG+GG | TC+CC | 473 | 226 | 0.750 | 0.143 | 2.116(0.776,5.770) | S:0.836(0.416,1.682) |
| rs32950 | rs863221 |  |  |  |  |  |  |
| AA | TT+GT | 14 | 8 |  |  |  |  |
| AA | GG | 84 | 59 | 0.642 | 0.235 | 1.901(0.659,5.485) | RERI:1.478(-2.547,5.504) |
| AG+GG | TT+GT | 721 | 332 | 0.3 | 0.558 | 1.350(0.495,3.684) | AP:0.396(-0.306,1.099) |
| AG+GG | GG | 8 | 6 | 1.316 | 0.126 | 3.729(0.692,20.093) | S:2.183(0.334,14.248) |
| rs33002 | rs1981928 |  |  |  |  |  |  |
| AA | TA+AA | 247 | 144 |  |  |  |  |
| AA | TT | 37 | 22 | -0.023 | 0.951 | 0.977(0.472,2.022) | RERI:-0.210(-0.861,0.442) |
| AT+TT | TA+AA | 481 | 223 | -0.134 | 0.507 | 0.874(0.588,1.300) | AP:-0.327(-1.387,0.734) |
| AT+TT | TT | 68 | 29 | -0.443 | 0.205 | 0.642(0.324,1.274) | S:2.416(0.033,176.649) |
| rs33002 | rs12999145 |  |  |  |  |  |  |
| AA | AG+GG | 208 | 116 |  |  |  |  |
| AA | AA | 78 | 50 | 0.262 | 0.362 | 1.300(0.739,2.284) | RERI:-0.126(-0.754,0.503) |
| AT+TT | AG+GG | 423 | 187 | -0.15 | 0.485 | 0.861(0.565,1.311) | AP:-0.121(-0.737,0.494) |
| AT+TT | AA | 131 | 66 | 0.034 | 0.909 | 1.035(0.578,1.854) | S:0.216(0.000,5194.667) |
| rs33002 | rs1042821 |  |  |  |  |  |  |
| AA | GA+AA | 132 | 59 |  |  |  |  |
| AA | GG | 146 | 105 | 0.305 | 0.210 | 1.356(0.842.2.185) | RERI:-0.277(-0.874,0.320) |
| AT+TT | GA+AA | 229 | 100 | -0.042 | 0.870 | 0.958(0.576,1.595) | AP:-0.266(-0.810,0.277) |
| AT+TT | GG | 315 | 153 | 0.038 | 0.881 | 1.039(0.630,1.714) | S:0.123(0.000,342.494) |
| rs33002 | rs2348244 |  |  |  |  |  |  |
| AA | TT | 92 | 52 |  |  |  |  |
| AA | TC+CC | 190 | 114 | 0.382 | 0.136 | 1.466(0.887,2.422) | RERI:-0.370(-1.039,0.300) |
| AT+TT | TT | 196 | 80 | 0.026 | 0.927 | 1.027(0.585,1.802) | AP:-0.330(-0.879,0.220) |
| AT+TT | TC+CC | 356 | 170 | 0.115 | 0.673 | 1.122(0.658,1.911) | S:0.248(0.023,2.688) |
| rs33002 | rs3776968 |  |  |  |  |  |  |
| AA | CT+TT | 39 | 11 |  |  |  |  |
| AA | CC | 247 | 154 | 0.879 | **0.047** | 2.409(1.012,5.732) | RERI:-0.992(-2.502,0.517) |
| AT+TT | CT+TT | 397 | 170 | 0.299 | 0.467 | 1.348(0.602,3.015) | AP:-0.562(-1.197,0.072) |
| AT+TT | CC | 153 | 83 | 0.568 | 0.217 | 1.765(0.716,4.353) | S:0.435(0.219,0.865) |
| rs33002 | rs863221 |  |  |  |  |  |  |
| AA | TT+GT | 260 | 146 |  |  |  |  |
| AA | GG | 23 | 19 | 1.078 | 0.062 | 2.938(0.947,9.115) | RERI:-1.023(-2.979,0.933) |
| AT+TT | TT+GT | 475 | 203 | -0.1 | 0.640 | 0.904(0.594,1.378) | AP:-0.562(-1.706,0.582) |
| AT+TT | GG | 73 | 46 | 0.599 | 0.179 | 1.821(0.760,4.364) | S:0.445(0.125,1.580) |
| rs33002 | rs33008 |  |  |  |  |  |  |
| AA | GC+CC | 29 | 5 |  |  |  |  |
| AA | GG | 257 | 161 | 0.674 | 0.252 | 1.961(0.619,6.217) | RERI:-2.167(-5.252,0.918) |
| AT+TT | GC+CC | 369 | 164 | 0.929 | 0.082 | 2.532(0.887,7.224) | AP:-1.633(-2.720,-0.546) |
| AT+TT | GG | 178 | 88 | 0.283 | 0.633 | 1.326(0.416,4.226) | S:0.131(0.0120,1.430) |
| rs2348244 | rs33008 |  |  |  |  |  |  |
| TT | GC+CC | 143 | 60 |  |  | 1 |  |
| TT | GG | 145 | 72 | -0.789 | **0.031** | 0.454(0.222,0.932) | RERI:0.379(0.049,0.708) |
| TC+CC | GC+CC | 255 | 107 | -0.121 | 0.597 | 0.886(0.566,1.387) | AP:0.527(-0.014,1.068) |
| TC+CC | GG | 286 | 178 | -0.330 | 0.351 | 0.719(0.360,1.437) | S:0.426(0.230,0.788) |
| Abbreviations: HC, healthy control; HCC, hepatocellular carcinoma; RERI, Relative Excess Risk of Interaction; AP, Attributable Proportion of interaction; S, Synergy index.When calculating covariance matrix, take SNPs other than the analysis SNPs, together with gender, age, drinking and smoking as control variables.The bold font shows statistical significance. | | | | | | | |

| Supplementary table 8 : Haplotype analysis for 13 SNPs near *MSH3* gene in NC vs. HCC, CHB vs. HCC, and LC vs HCC groups by Haploview 4.2. | | | | | |
| --- | --- | --- | --- | --- | --- |
| Group | Haplotype | Freq. | Case, Control Ratios | *P* value | OR(95%CI) |
| NC vs. HCC |  |  |  |  |  |
|  | Block 1 |  |  |  |  |
|  | ACCC | 0.361 | 311.0 : 527.0, 350.0 : 642.0 | 0.4164 | 1.082(0.894,1.311) |
|  | GCTT | 0.346 | 289.5 : 548.5, 344.4 : 647.6 | 0.9404 | 0.995(0.820,1.207) |
|  | GTTT | 0.252 | 207.4 : 630.6, 253.3 : 738.7 | 0.6977 | 0.958(0.775,1.185) |
|  | GCTC | 0.035 | 27.1 : 810.9, 36.2 : 955.8 | 0.6247 | 0.884(0.532,1.469) |
|  | Block 2 |  |  |  |  |
|  | GG | 0.624 | 525.0 : 313.0, 614.0 : 374.0 | 0.8249 | 1.022(0.845,1.236) |
|  | AA | 0.376 | 313.0 : 525.0, 374.0 : 614.0 | 0.8249 | 0.979(0.809,1.184) |
|  | Block 3 |  |  |  |  |
|  | CGG | 0.392 | 331.0 : 509.0, 387.7 : 604.3 | 0.8877 | 1.012(0.839,1.222) |
|  | TTG | 0.280 | 234.6 : 605.4, 278.4 : 713.6 | 0.9495 | 0.998(0.813,1.224) |
|  | CGC | 0.236 | 193.1 : 646.9, 240.1 : 751.9 | 0.5410 | 0.935(0.753,1.161) |
|  | TGG | 0.086 | 76.8 : 763.2, 79.9 : 912.1 | 0.4070 | 1.150(0.829,1.596) |
|  | Block 4 |  |  |  |  |
|  | AAA | 0.379 | 316.9 : 523.1, 377.8 : 614.2 | 0.8762 | 0.985(0.815,1.190) |
|  | TAT | 0.373 | 317.6 : 522.4, 366.0 : 626.0 | 0.6865 | 1.042(0.862,1.260) |
|  | AGT | 0.237 | 199.4 : 640.6, 234.0 : 758.0 | 0.9407 | 1.006(0.810,1.248) |
| CHB vs. HCC |  |  |  |  |  |
|  | Block 1 |  |  |  |  |
|  | ACCC | 0.359 | 311.0 : 527.0, 477.9 : 882.1 | 0.3492 | 1.089(0.910,1.302) |
|  | GCTT | 0.356 | 289.6 : 548.4, 493.6 : 866.4 | 0.4085 | 0.928(0.775,1.111) |
|  | GTTT | 0.248 | 208.2 : 629.8, 336.8 : 1023.2 | 0.9664 | 1.002(0.821,1.223) |
|  | GCTC | 0.034 | 26.2 : 811.8, 47.7 : 1312.3 | 0.6327 | 0.875(0.539,1.422) |
|  | Block 2 |  |  |  |  |
|  | GG | 0.615 | 525.0 : 313.0, 831.0 : 535.0 | 0.3953 | 1.080(0.905,1.289) |
|  | AA | 0.385 | 313.0 : 525.0, 535.0 : 831.0 | 0.3953 | 0.926(0.776,1.106) |
|  | Block 3 |  |  |  |  |
|  | CGG | 0.380 | 330.2 : 509.8, 509.5 : 858.5 | 0.3309 | 1.090(0.913,1.300) |
|  | TTG | 0.297 | 234.7 : 605.3, 421.3 : 946.7 | 0.1532 | 0.874(0.723,1.056) |
|  | CGC | 0.232 | 193.9 : 646.1, 318.7 : 1049.3 | 0.9073 | 0.988(0.806,1.211) |
|  | TGG | 0.087 | 77.6 : 762.4, 115.4 : 1252.6 | 0.5183 | 1.115(0.825,1.507) |
|  | Block 4 |  |  |  |  |
|  | TAT | 0.370 | 317.6 : 522.4, 498.6 : 865.4 | 0.5558 | 1.056(0.884,1.261) |
|  | AAA | 0.370 | 316.9 : 523.1, 497.9 : 866.1 | 0.5631 | 1.054(0.882,1.259) |
|  | AGT | 0.247 | 199.4 : 640.6, 345.2 : 1018.8 | 0.4057 | 0.917(0.750,1.121) |
| LC vs. HCC |  |  |  |  |  |
|  | Block 1 |  |  |  |  |
|  | ACCC | 0.361 | 312.0 : 528.0, 474.8 : 867.2 | 0.4044 | 1.079(0.902,1.290) |
|  | GCTT | 0.345 | 289.5 : 550.5, 462.8 : 879.2 | 0.9949 | 0.999(0.833,1.198) |
|  | GTTT | 0.265 | 207.4 : 632.6, 371.1 : 970.9 | 0.1267 | 0.856(0.703,1.043) |
|  | GCTC | 0.025 | 27.0 : 813.0, 28.3 : 1313.7 | 0.1076 | 1.559(0.912,2.663) |
|  | Block 2 |  |  |  |  |
|  | GG | 0.634 | 525.0 : 313.0, 859.0 : 487.0 | 0.5812 | 0.951(0.795,1.137) |
|  | AA | 0.366 | 313.0 : 525.0, 487.0 : 859.0 | 0.5812 | 1.052(0.879,1.257) |
|  | Block 3 |  |  |  |  |
|  | CGG | 0.380 | 330.3 : 509.7, 504.6 : 851.4 | 0.3228 | 1.090(0.914,1.301) |
|  | TTG | 0.286 | 234.6 : 605.4, 393.2 : 962.8 | 0.5895 | 0.952(0.786,1.152) |
|  | CGC | 0.244 | 193.7 : 646.3, 341.7 : 1014.3 | 0.2575 | 0.890(0.728,1.090) |
|  | TGG | 0.085 | 77.4 : 762.6, 110.1 : 1245.9 | 0.3695 | 1.143(0.843,1.551) |
|  | Block 4 |  |  |  |  |
|  | AAA | 0.370 | 316.9 : 523.1, 495.8 : 860.2 | 0.5838 | 1.051(0.880,1.256) |
|  | TAT | 0.370 | 317.5 : 522.5, 494.5 : 861.5 | 0.5296 | 1.059(0.886,1.265) |
|  | AGT | 0.246 | 199.4 : 640.6, 340.3 : 1015.7 | 0.4720 | 0.928(0.759,1.134) |
| Abbreviations: NC, natural clearance; CHB, chronic hepatitis B; LC, liver cirrhosis; HCC, hepatocellular carcinoma; SNPs,single nucleotide polymorphisms; *:*P*<0.05.  The blocks of haplotype analysis are the same in the three groups: Four SNPs of Block1, including rs1805355_G, rs3776968_T, rs1428030_C, rs181747_C; Three SNPs of Block 2, including rs32950_G, rs40139_G; Four SNPs of Block 3, including rs26779_T, rs12513549_T, rs33008_C; Three SNPs of Block 4, including rs33002_T, rs26279_A, rs2112416_A. | | | | | |

| Supplementary table 9 : The negative results of additive interaction analysis of SNPs between NC and HCC group. | | | | | | | |
| --- | --- | --- | --- | --- | --- | --- | --- |
| SNP1 | SNP2 | NC | HCC | B | *P* | OR(95%CI) | RERI/AP/S |
| rs4952887 | rs1042821 |  |  |  |  |  |  |
| TT+CT | GG+GA | 136 | 100 |  |  | 1 |  |
| TT+CT | AA | 4 | 4 | 0.653 | 0.435 | 1.921(0.374,9.879) | RERI:2.318(-2.11,6.746) |
| CC | GG+GA | 338 | 291 | 0.351 | 0.087 | 1.421(0.95,2.125) | AP:0.497(-0.192,1.187) |
| CC | AA | 10 | 21 | 1.539 | **0.002** | 4.658(1.729,12.552) | S:2.728(0.292,25.446) |
| rs4952887 | rs2348244 |  |  |  |  |  |  |
| TT+CT | TT | 54 | 27 |  |  | 1 |  |
| TT+CT | TC+CC | 90 | 76 | 0.484 | 0.140 | 1.622(0.854,3.083) | RERI:0.123(-0.745,0.99) |
| CC | TT | 116 | 104 | 0.431 | 0.193 | 1.539(0.804,2.945) | AP:0.054(-0.334,0.441) |
| CC | TC+CC | 234 | 208 | 0.826 | **0.009** | 2.284(1.23,4.24) | S:1.106(0.509,2.403) |
| rs4952887 | rs181747 |  |  |  |  |  |  |
| TT+CT | TT+TC | 116 | 81 |  |  | 1 |  |
| TT+CT | CC | 28 | 23 | 0.408 | 0.284 | 1.504(0.713,3.173) | RERI:-0.141(-1.302,1.02) |
| CC | TT+TC | 296 | 253 | 0.408 | 0.065 | 1.503(0.975,2.319) | AP:-0.076(-0.71,0.558) |
| CC | CC | 50 | 59 | 0.624 | **0.045** | 1.866(1.015,3.431) | S:0.86(0.258,2.868) |
| rs4952887 | rs40139 |  |  |  |  |  |  |
| TT+CT | AA+AG | 78 | 56 |  |  | 1 |  |
| TT+CT | GG | 66 | 48 | 0.211 | 0.501 | 1.235(0.668,2.283) | RERI:0.36(-0.385,1.105) |
| CC | AA+AG | 224 | 189 | 0.3 | 0.246 | 1.35(0.814,2.239) | AP:0.185(-0.195,0.565) |
| CC | GG | 120 | 124 | 0.665 | **0.023** | 1.945(1.095,3.453) | S:1.615(0.434,6.008) |
| rs4952887 | rs26779 |  |  |  |  |  |  |
| TT+CT | CC+CT | 126 | 82 |  |  | 1 |  |
| TT+CT | TT | 18 | 21 | 1.019 | 0.168 | 2.769(0.65,11.791) | RERI:0.34(-1.941,2.621) |
| CC | CC+CT | 304 | 261 | 0.389 | 0.070 | 1.475(0.968,2.248) | AP:0.095(-0.521,0.71) |
| CC | TT | 46 | 50 | 1.277 | 0.068 | 3.587(0.911,14.131) | S:1.151(0.436,3.041) |
| rs1981928 | rs13019654 |  |  |  |  |  |  |
| AA | TT | 40 | 22 |  |  | 1 |  |
| AA | GG+GT | 172 | 142 | 0.483 | 0.158 | 1.621(0.829,3.169) | RERI:0.425(-0.13,0.981) |
| TT+TA | TT | 0 | 0 | 0 | 0 | 1 | AP:0.208(-0.017,0.433) |
| TT+TA | GG+GT | 276 | 252 | 0.716 | **0.036** | 2.047(1.049,3.996) | S:1.685(0.79,3.592) |
| rs1981928 | rs1042821 |  |  |  |  |  |  |
| AA | GG+GA | 202 | 151 |  |  | 1 |  |
| AA | AA | 10 | 10 | 0.509 | 0.342 | 1.663(0.583,4.745) | RERI:5.172(-2.769,13.113) |
| TT+TA | GG+GA | 268 | 241 | 0.182 | 0.390 | 1.2(0.792,1.817) | AP:0.73(0.375,1.095) |
| TT+TA | AA | 4 | 15 | 1.951 | **0.003** | 7.037(1.904,26.006) | S:6.992(0.787,62.079) |
| rs1981928 | rs2348244 |  |  |  |  |  |  |
| AA | TT | 68 | 56 |  |  | 1 |  |
| AA | TC+CC | 146 | 107 | 0.126 | 0.644 | 1.135(0.664,1.939) | RERI:0.625(0.089,1.161) |
| TT+TA | TT | 100 | 76 | -0.095 | 0.762 | 0.909(0.492,1.682) | AP:0.375(0.033,0.716) |
| TT+TA | TC+CC | 176 | 177 | 0.512 | 0.084 | 1.668(0.933,2.982) | S:15.316(0,1157820789.8) |
| rs1981928 | rs181747 |  |  |  |  |  |  |
| AA | TT+TC | 180 | 131 |  |  | 1 |  |
| AA | CC | 30 | 32 | 0.226 | 0.508 | 1.254(0.641,2.453) | RERI:0.191(-0.388,0.771) |
| TT+TA | TT+TC | 228 | 204 | 0.221 | 0.315 | 1.247(0.811,1.917) | AP:0.113(-0.253,0.479) |
| TT+TA | CC | 48 | 50 | 0.526 | 0.105 | 1.692(0.897,3.193) | S:1.382(0.361,5.294) |
| rs1981928 | rs863221 |  |  |  |  |  |  |
| AA | TT+GT | 168 | 137 |  |  | 1 |  |
| AA | GG | 34 | 25 | -0.56 | 0.434 | 0.571(0.14,2.325) | RERI:0.052(-0.545,0.649) |
| TT+TA | TT+GT | 244 | 213 | 0.208 | 0.338 | 1.231(0.805,1.885) | AP:0.061(-0.622,0.744) |
| TT+TA | GG | 28 | 40 | -0.157 | 0.821 | 0.855(0.22,3.326) | S:0.735(0.022,24.261) |
| rs1981928 | rs26779 |  |  |  |  |  |  |
| AA | CC+CT | 176 | 135 |  |  | 1 |  |
| AA | TT | 38 | 27 | 0.779 | 0.259 | 2.18(0.563,8.448) | RERI:1.248(-0.826,3.322) |
| TT+TA | CC+CT | 250 | 209 | 0.19 | 0.383 | 1.209(0.789,1.852) | AP:0.343(-0.099,0.785) |
| TT+TA | TT | 26 | 44 | 1.291 | 0.078 | 3.638(0.865,15.294) | S:1.899(0.658,5.483) |
| rs13019654 | rs2303428 |  |  |  |  |  |  |
| TT | CC | 2 | 3 |  |  | 1 |  |
| TT | TT+TC | 39 | 19 | -0.852 | 0.403 | 0.426(0.058,3.148) | RERI:0.76(0.196,1.323) |
| GG+GT | CC | 54 | 38 | -0.505 | 0.612 | 0.603(0.085,4.256) | AP:0.962(-1.345,3.268) |
| GG+GT | TT+TC | 391 | 354 | -0.236 | 0.810 | 0.79(0.116,5.382) | S:0.217(0.002,28.173) |
| rs13019654 | rs181747 |  |  |  |  |  |  |
| TT | TT+TC | 34 | 17 |  |  | 1 |  |
| TT | CC | 6 | 5 | 0.317 | 0.670 | 1.374(0.319,5.909) | RERI:0.137(-1.721,1.994) |
| GG+GT | TT+TC | 378 | 317 | 0.493 | 0.196 | 1.637(0.776,3.454) | AP:0.064(-0.805,0.932) |
| GG+GT | CC | 72 | 76 | 0.764 | 0.078 | 2.147(0.918,5.022) | S:1.135(0.178,7.26) |
| rs13019654 | rs40139 |  |  |  |  |  |  |
| TT | AA+AG | 28 | 14 |  |  | 1 |  |
| TT | GG | 10 | 8 | 1.236 | 0.086 | 3.441(0.84,14.089) | RERI:-1.797(-5.53,1.936) |
| GG+GT | AA+AG | 272 | 231 | 0.741 | 0.063 | 2.098(0.96,4.586) | AP:-0.655(-1.904,0.594) |
| GG+GT | GG | 176 | 163 | 1.009 | **0.015** | 2.743(1.214,6.201) | S:0.492(0.187,1.3) |
| rs13019654 | rs863221 |  |  |  |  |  |  |
| TT | TT+GT | 32 | 18 |  |  | 1 |  |
| TT | GG | 8 | 4 | -0.366 | 0.726 | 0.694(0.09,5.347) | RERI:-0.287(-1.49,0.916) |
| GG+GT | TT+GT | 386 | 332 | 0.497 | 0.182 | 1.643(0.793,3.406) | AP:-0.273(-1.367,0.82) |
| GG+GT | GG | 54 | 61 | 0.049 | 0.948 | 1.05(0.241,4.58) | S:0.149(0,13732.284) |
| rs13019654 | rs26779 |  |  |  |  |  |  |
| TT | CC+CT | 34 | 17 |  |  | 1 |  |
| TT | TT | 8 | 5 | 1.163 | 0.246 | 3.201(0.448,22.863) | RERI:0.294(-3.611,4.198) |
| GG+GT | CC+CT | 396 | 326 | 0.532 | 0.158 | 1.702(0.814,3.558) | AP:0.07(-0.857,0.997) |
| GG+GT | TT | 56 | 66 | 1.434 | 0.053 | 4.196(0.983,17.91) | S:1.101(0.289,4.2) |
| rs2303428 | rs1042821 |  |  |  |  |  |  |
| CC | GG+GA | 54 | 36 |  |  | 1 |  |
| CC | AA | 4 | 3 | 0.534 | 0.582 | 1.706(0.255,11.404) | RERI:1.853(-1.946,5.653) |
| TT+TC | GG+GA | 414 | 354 | 0.147 | 0.605 | 1.159(0.663,2.027) | AP:0.499(-0.294,1.291) |
| TT+TC | AA | 10 | 22 | 1.313 | **0.010** | 3.716(1.362,10.137) | S:3.145(0.123,80.362) |
| rs2303428 | rs40139 |  |  |  |  |  |  |
| CC | AA+AG | 36 | 23 |  |  | 1 |  |
| CC | GG | 22 | 18 | 0.809 | 0.105 | 2.246(0.844,5.975) | RERI:-0.778(-2.537,0.981) |
| TT+TC | AA+AG | 266 | 222 | 0.415 | 0.245 | 1.514(0.753,3.047) | AP:-0.393(-1.22,0.435) |
| TT+TC | GG | 158 | 154 | 0.684 | 0.075 | 1.981(0.933,4.206) | S:0.558(0.22,1.414) |
| rs2303428 | rs863221 |  |  |  |  |  |  |
| CC | TT+GT | 44 | 36 |  |  | 1 |  |
| CC | GG | 10 | 5 | -1.197 | 0.214 | 0.302(0.046,2) | RERI:0.375(-0.156,0.906) |
| TT+TC | TT+GT | 371 | 312 | 0.071 | 0.810 | 1.074(0.6,1.921) | AP:0.499(-0.281,1.28) |
| TT+TC | GG | 51 | 60 | -0.287 | 0.686 | 0.751(0.187,3.016) | S:0.4(0.108,1.475) |
| rs1042821 | rs40139 |  |  |  |  |  |  |
| GG+GA | AA+AG | 292 | 226 |  |  | 1 |  |
| GG+GA | GG | 176 | 166 | 0.364 | 0.057 | 1.44(0.99,2.094) | RERI:-2.206(-6.232,1.82) |
| AA | AA+AG | 8 | 18 | 1.358 | **0.006** | 3.889(1.474,10.262) | AP:-1.04(-3.74,1.66) |
| AA | GG | 6 | 7 | 0.752 | 0.281 | 2.122(0.541,8.324) | S:0.337(0.034,3.299) |
| rs2348244 | rs181747 |  |  |  |  |  |  |
| TT | TT+TC | 138 | 105 |  |  | 1 |  |
| TT | CC | 28 | 26 | 0.294 | 0.434 | 1.341(0.643,2.799) | RERI:0.126(-0.976,1.228) |
| TC+CC | TT+TC | 274 | 229 | 0.424 | **0.030** | 1.529(1.042,2.244) | AP:0.063(-0.476,0.603) |
| TC+CC | CC | 50 | 56 | 0.691 | **0.018** | 1.996(1.125,3.54) | S:1.145(0.341,3.844) |
| rs2348244 | rs40139 |  |  |  |  |  |  |
| TT | AA+AG | 106 | 71 |  |  | 1 |  |
| TT | GG | 60 | 61 | 0.717 | **0.017** | 2.049(1.135,3.698) | RERI:-0.774(-1.901,0.352) |
| TC+CC | AA+AG | 196 | 174 | 0.664 | **0.004** | 1.943(1.237,3.053) | AP:-0.349(-0.867,0.169) |
| TC+CC | GG | 126 | 111 | 0.796 | **0.003** | 2.216(1.315,3.736) | S:0.611(0.33,1.13) |
| rs2348244 | rs26779 |  |  |  |  |  |  |
| TT | CC+CT | 146 | 113 |  |  | 1 |  |
| TT | TT | 24 | 17 | 0.785 | 0.272 | 2.192(0.541,8.891) | RERI:1.416(-0.746,3.578) |
| TC+CC | CC+CT | 284 | 230 | 0.386 | **0.043** | 1.471(1.013,2.135) | AP:0.347(-0.088,0.782) |
| TC+CC | TT | 40 | 54 | 1.406 | **0.044** | 4.079(1.039,16.016) | S:1.851(0.671,5.104) |
| rs181747 | rs40139 |  |  |  |  |  |  |
| TT+TC | AA+AG | 288 | 231 |  |  | 1 |  |
| TT+TC | GG | 118 | 105 | 0.356 | 0.076 | 1.428(0.964,2.116) | RERI:-0.28(-1.719,1.158) |
| CC | AA+AG | 12 | 15 | 0.484 | 0.314 | 1.622(0.633,4.156) | AP:-0.158(-0.991,0.675) |
| CC | GG | 66 | 67 | 0.571 | **0.015** | 1.77(1.117,2.805) | S:0.733(0.171,3.149) |
| rs863221 | rs26779 |  |  |  |  |  |  |
| TT+GT | CC+CT | 414 | 342 |  |  | 1 |  |
| TT+GT | TT | 4 | 7 | 1.144 | 0.117 | 3.14(0.751,13.136) | RERI:-1.935(-7.088,3.219) |
| GG | CC+CT | 2 | 1 | 0.332 | 0.791 | 1.393(0.119,16.243) | AP:-1.21(-4.502,2.081) |
| GG | TT | 60 | 64 | 0.469 | 0.054 | 1.598(0.992,2.575) | S:0.236(0.025,2.202) |
| rs33002 | rs1981928 |  |  |  |  |  |  |
| AA | AA | 92 | 68 |  |  |  |  |
| AA | TT+TA | 102 | 98 | 0.62 | **0.027** | 1.858(1.075,3.212) | RERI:-1.061(-2.124,0.001) |
| AT+TT | AA | 122 | 95 | 0.708 | **0.010** | 2.029(1.182,3.482) | AP:-0.581(-1.137,-0.024) |
| AT+TT | TT+TA | 174 | 157 | 0.603 | **0.036** | 1.828(1.041,3.210) | S:0.438(0.236,0.812) |
| rs33002 | rs13019654 |  |  |  |  |  |  |
| AA | TT | 18 | 8 |  |  |  |  |
| AA | GG+GT | 180 | 157 | 0.784 | 0.125 | 2.191(0.804,5.970) | RERI:-0.376(-2.265,1.514) |
| AT+TT | TT | 24 | 14 | 0.72 | 0.264 | 2.054(0.581,7.260) | AP:-0.131(-0.736,0.474) |
| AT+TT | GG+GT | 272 | 236 | 1.054 | **0.048** | 2.869(1.011,8.141) | S:0.833(0.395,1.754) |
| rs33002 | rs2303428 |  |  |  |  |  |  |
| AA | CC | 26 | 14 |  |  |  |  |
| AA | TT+TC | 168 | 151 | 0.757 | 0.080 | 2.132(0.913,4.978) | RERI:-1.495(-3.820,0.830) |
| AT+TT | CC | 32 | 27 | 1.099 | **0.035** | 3.001(1.082,8.324) | AP:-0.567(-1.277,0.143) |
| AT+TT | TT+TC | 262 | 225 | 0.97 | **0.034** | 2.637(1.078,6.448) | S:0.523(0.292,0.938) |
| rs33002 | rs1042821 |  |  |  |  |  |  |
| AA | GG+GA | 192 | 158 |  |  |  |  |
| AA | AA | 6 | 6 | 0.089 | 0.899 | 1.093(0.277,4.309) | RERI:4.322(-0.584,9.227) |
| AT+TT | GG+GA | 284 | 234 | 0.24 | 0.242 | 1.271(0.851,1.898) | AP:0.760(0.461,1.059) |
| AT+TT | AA | 8 | 19 | 1.738 | **0.001** | 5.687(1.980,16.336) | S:12.862(0.300,551.621) |
| rs33002 | rs2348244 |  |  |  |  |  |  |
| AA | TT | 78 | 52 |  |  |  |  |
| AA | TC+CC | 118 | 114 | 0.613 | **0.021** | 1.846(1.096,3.110) | RERI:-0.333(-1.285,0.618) |
| AT+TT | TT | 92 | 80 | 0.533 | 0.082 | 1.705(0.935,3.110) | AP:-0.150(-0.573,0.272) |
| AT+TT | TC+CC | 206 | 170 | 0.796 | **0.004** | 2.217(1.289,3.813) | S:0.785(0.428,1.438) |
| rs33002 | rs181747 |  |  |  |  |  |  |
| AA | TT+TC | 122 | 85 |  |  |  |  |
| AA | CC | 76 | 81 | 0.471 | 0.100 | 1.601(0.914,2.805) | RERI:1.346(-6.556,9.248) |
| AT+TT | TT+TC | 292 | 250 | 0.29 | 0.155 | 1.337(0.896,1.995) | AP:0.410(-1.0190,1.839) |
| AT+TT | CC | 2 | 1 | 1.189 | 0.453 | 3.285(0.147,73.249) | S:2.435(0.0712,83.173) |
| rs33002 | rs40139 |  |  |  |  |  |  |
| AA | AA+AG | 116 | 91 |  |  |  |  |
| AA | GG | 78 | 75 | -0.088 | 0.820 | 0.916(0.428,1.957) | RERI:0.664(0.029,1.300) |
| AT+TT | AA+AG | 186 | 154 | 0.224 | 0.296 | 1.25(0.823,1.901) | AP:0.363(0.051,0.675) |
| AT+TT | GG | 108 | 98 | 0.605 | **0.014** | 1.83(1.129,2.967) | S:4.983(0.108,229.755) |
| rs33002 | rs26779 |  |  |  |  |  |  |
| AA | CC+CT | 174 | 142 |  |  |  |  |
| AA | TT | 24 | 22 | 0.962 | 0.176 | 2.617(0.649,10.546) | RERI:0.445(-1.651,2.541) |
| AT+TT | CC+CT | 258 | 201 | 0.307 | 0.178 | 1.359(0.870,2.122) | AP:0.130(-0.450,0.710) |
| AT+TT | TT | 40 | 49 | 1.23 | 0.076 | 3.421(0.877,13.340) | S:1.225(0.459,3.273) |
| Abbreviations: NC, natural clearance; HCC, hepatocellular carcinoma; RERI, Relative Excess Risk of Interaction; AP, Attributable Proportion of interaction; S, Synergy index. When calculating covariance matrix, take SNPs other than the analysis SNPs, together with gender, age, drinking and smoking as control variables. The bold font shows statistical significance. | | | | | | | |

| Supplementary table 10 : The negative results of additive interaction analysis of SNPs between CHB and HCC group. | | | | | | | |
| --- | --- | --- | --- | --- | --- | --- | --- |
| SNP1 | SNP2 | CHB | HCC | B | *P* | OR(95%CI) | RERI/AP/S |
| rs181747 | rs12999145 |  |  |  |  |  |  |
| TT＋TC | AA＋AG | 459 | 256 |  |  | 1.0 |  |
| TT＋TC | GG | 121 | 80 | 0.351 | 0.189 | 1.421(0.841,2.399) | RERI:-0.871(-1.899,0.157) |
| CC | AA＋AG | 77 | 67 | 0.444 | 0.137 | 1.559(0.868,2.800) | AP:-0.786(-2.126,0.555) |
| CC | GG | 20 | 15 | 0.103 | 0.836 | 1.108(0.420,2.924) | S:0.111(0.000,117.628) |
| rs181747 | rs4952887 |  |  |  |  |  |  |
| TT＋TC | CT＋TT | 170 | 81 |  |  | 1.0 |  |
| TT＋TC | CC | 406 | 253 | 0.245 | 0.220 | 1.278(0.863,1.893) | RERI:-0.372(-1.518,0.773) |
| CC | CT＋TT | 31 | 23 | 0.521 | 0.199 | 1.684(0.760,3.730) | AP:-0.234(-0.983,0.514) |
| CC | CC | 66 | 59 | 0.463 | 0.178 | 1.589(0.809,3.117) | S:0.613(0.157,2.385) |
| rs181747 | rs2303428 |  |  |  |  |  |  |
| TT＋TC | CC | 61 | 33 |  |  | 1.0 |  |
| TT＋TC | TT＋TC | 519 | 302 | 0.298 | 0.350 | 1.348(0.721,2.521) | RERI:0.462(-0.728,1.652) |
| CC | CC | 10 | 8 | 0.068 | 0.918 | 1.071(0.293,3.910) | AP:0.246(-0.383,0.875) |
| CC | TT＋TC | 86 | 73 | 0.631 | 0.131 | 1.880(0.829,4.265) | S:2.106(0.110,40.424) |
| rs32950 | rs12999145 |  |  |  |  |  |  |
| AG＋GG | AA＋AG | 442 | 260 |  |  | 1.0 |  |
| AA | GG | 120 | 80 | 0.245 | 0.355 | 1.278(0.760,2.150) | RERI:-0.016(-1.389,1.356) |
| AG＋GG | AA＋AG | 87 | 55 | 0.357 | 0.119 | 1.429(0.912,2.237) | AP:-0.010(-0.828,0.808) |
| AA | GG | 19 | 12 | 0.525 | 0.287 | 1.690(0.644,4.437) | S:0.977(0.135,7.060) |
| rs32950 | rs1981928 |  |  |  |  |  |  |
| AG＋GG | AA | 209 | 132 |  |  | 1.0 |  |
| AA | TT＋TA | 352 | 207 | 0.177 | 0.429 | 1.193(0.770,1.850) | RERI:-0.100(-1.136,0.936) |
| AG＋GG | AA | 41 | 25 | 0.417 | 0.206 | 1.517(0.795,2.896) | AP:-0.062(-0.717,0.593) |
| AA | TT＋TA | 65 | 42 | 0.477 | 0.133 | 1.612(0.865,3.002) | S:0.860(0.185,3.989) |
| rs32950 | rs4952887 |  |  |  |  |  |  |
| AG＋GG | CT＋TT | 172 | 85 |  |  | 1.0 |  |
| AA | CC | 387 | 253 | 0.203 | 0.307 | 1.226(0.829,1.812) | RERI:-0.022(-1.123,1.080) |
| AG＋GG | CT＋TT | 30 | 19 | 0.398 | 0.292 | 1.489(0.710,3.124) | AP:-0.013(-0.666,0.640) |
| AA | CC | 76 | 48 | 0.526 | 0.068 | 1.692(0.961,2.979) | S:0.970(0.205,4.583) |
| rs32950 | rs2303428 |  |  |  |  |  |  |
| AG＋GG | CC | 61 | 34 |  |  | 1.0 |  |
| AA | TT＋TC | 501 | 305 | 0.438 | 0.159 | 1.549(0.843,2.846) | RERI:-1.458(-5.015,2.100) |
| AG＋GG | CC | 8 | 5 | 1.093 | 0.136 | 2.983(0.710,12.541) | AP:-0.702(-2.412,1.007) |
| AA | TT＋TC | 98 | 62 | 0.730 | **0.046** | 2.074(1.012,4.249) | S:0.424(0.099,1.817) |
| rs26779 | rs12999145 |  |  |  |  |  |  |
| CT＋TT | AA＋AG | 350 | 180 |  |  | 1.0 |  |
| CC | GG | 83 | 61 | 0.431 | 0.141 | 1.539(0.867,2.730) | RERI:-0.635(-1.550,0.279) |
| CT＋TT | AA＋AG | 187 | 141 | 0.428 | 0.333 | 1.534(0.646,3.643) | AP:-0.442(-1.200,0.316) |
| CC | GG | 58 | 34 | 0.363 | 0.493 | 1.437(0.509,4.061) | S:0.408(0.090,1.849） |
| rs26779 | rs1981928 |  |  |  |  |  |  |
| CT＋TT | AA | 152 | 91 |  |  | 1.0 |  |
| CC | TT＋TA | 279 | 150 | 0.056 | 0.820 | 1.057(0.654,1.709) | RERI:0.344(-0.252,0.940) |
| CT＋TT | AA | 101 | 71 | 0.166 | 0.727 | 1.180(0.465,2.996) | AP:0.218(-0.148,0.583) |
| CC | TT＋TA | 146 | 103 | -0.459 | 0.344 | 1.583(0.612,8.098) | S:2.446(0.189,31.693) |
| rs26779 | rs4952887 |  |  |  |  |  |  |
| CT＋TT | CT＋TT | 134 | 56 |  |  | 1.0 |  |
| CC | CC | 297 | 184 | 0.346 | 0.130 | 1.413(0.903,2.210) | RERI:-0.491(-1.420,0.438) |
| CT＋TT | CT＋TT | 71 | 47 | 0.565 | 0.243 | 1.760(0.682,4.545) | AP:-0.292(-0.839,0.255) |
| CC | CC | 175 | 127 | 0.520 | 0.261 | 1.682(0.679,4.171) | S:0.582(0.253,1.338) |
| rs26779 | rs2303428 |  |  |  |  |  |  |
| CT＋TT | CC | 38 | 24 |  |  | 1.0 |  |
| CC | TT＋TC | 394 | 216 | 0.155 | 0.672 | 1.167(0.571,2.387) | RERI:0.593(0.014,1.171) |
| CT＋TT | CC | 32 | 17 | -0.076 | 0.902 | 0.927(0.276,3.117) | AP:0.351(0.013,0.690) |
| CC | TT＋TC | 211 | 156 | 0.523 | 0.335 | 1.687(0.582,4.884) | S:7.273(0.004,13142.587) |
| rs863221 | rs12999145 |  |  |  |  |  |  |
| TG＋GG | AA＋AG | 330 | 174 |  |  | 1.0 |  |
| TT | GG | 79 | 57 | 0.371 | 0.211 | 1.449(0.811,2.588) | RERI:-0.399(-1.399,0.0602) |
| TG＋GG | AA＋AG | 202 | 147 | 0.051 | 0.910 | 1.052(0.435,2.548) | AP:-0.361(-1.170,0.447) |
| TT | GG | 62 | 38 | 0.098 | 0.851 | 1.103(0.397,3.063) | S:0.205(0.004,10.072) |
| rs863221 | rs4952887 |  |  |  |  |  |  |
| TG＋GG | CT＋TT | 122 | 52 |  |  | 1.0 |  |
| TT | CC | 284 | 178 | 0.356 | 0.127 | 1.427(0.903,2.255) | RERI:-0.456(-1.219,0.288) |
| TG＋GG | CT＋TT | 79 | 51 | 0.214 | 0.661 | 1.239(0.476,3.224) | AP:-0.388(-0.985,0.210) |
| TT | CC | 184 | 133 | 0.183 | 0.711 | 1.201(0.455,3.166) | S:0.301,(0.056,1.609) |
| rs863221 | rs2303428 |  |  |  |  |  |  |
| TG＋GG | CC | 37 | 21 |  |  | 1.0 |  |
| TT | TT＋TC | 372 | 209 | 0.339 | 0.373 | 1.403(0.666,2.959) | RERI:0.004(-0.618,0.627) |
| TG＋GG | CC | 34 | 20 | -0.044 | 0.944 | 0.957(0.283,3.234) | AP:0.003(-0.453,0.460) |
| TT | TT＋TC | 231 | 163 | 0.311 | 0.583 | 1.365(0.449,4.149) | S:1.012(0.179,5.706) |
| rs12999145 | rs1981928 |  |  |  |  |  |  |
| AA＋AG | AA | 117 | 73 |  |  | 1.0 |  |
| GG | TT＋TA | 422 | 252 | 0.173 | 0.423 | 1.188(0.779,1.813) | RERI:-0.409(-1.486,0.668) |
| AA＋AG | AA | 137 | 91 | 0.255 | 0.328 | 1.290(0.774,2.149) | AP:-0.382(-1.276,0.512) |
| GG | TT＋TA | 4 | 4 | 0.068 | 0.943 | 1.070(0.168,6.818) | S:0.147(0.000,83.838) |
| rs12999145 | rs2303428 |  |  |  |  |  |  |
| AA＋AG | CC | 10 | 4 |  |  | 1.0 |  |
| GG | TT＋TC | 61 | 37 | 0.243 | 0.740 | 1.275(0.304,5.351) | RERI:0.224(-0.941,1.389) |
| AA＋AG | CC | 529 | 329 | 0.122 | 0.874 | 1.129(0.251,5.080) | AP:0.138(-0.689,0.964) |
| GG | TT＋TC | 81 | 57 | 0.488 | 0.517 | 1.630(0.372,7.142) | S:1.554(0.024,100.046) |
| rs1981928 | rs2303428 |  |  |  |  |  |  |
| AA | CC | 65 | 39 |  |  | 1.0 |  |
| TT＋TA | TT＋TC | 189 | 123 | 0.319 | 0.300 | 1.375(0.753,2.513) | RERI:0.415(-1.016,1.846） |
| AA | CC | 5 | 2 | -0.190 | 0.852 | 0.827(0.113,6.055) | AP:0.257（-0.644,1.158） |
| TT＋TA | TT＋TC | 419 | 252 | 0.481 | 0.182 | 1.618(0.798,3.282) | S:3.047（0.002,6155.287） |
| rs33002 | rs32950 |  |  |  |  |  |  |
| AA | AG＋GG | 205 | 136 |  |  |  |  |
| AA | AA | 55 | 25 | 0.109 | 0.740 | 1.116(0.585,2.128) | RERI:0.561(-0.341,1.463) |
| AT+TT | AG＋GG | 355 | 203 | 0.034 | 0.876 | 1.035(0.672,1.593) | AP:0.328(-0.117,0.773) |
| AT+TT | AA | 51 | 42 | 0.537 | 0.073 | 1.712(0.951,3.081) | S:4.747(0.047,483.915) |
| rs33002 | rs26779 |  |  |  |  |  |  |
| AA | CT＋TT | 172 | 82 |  |  |  |  |
| AA | CC | 92 | 82 | 0.643 | 0.235 | 1.901(0.659,5.487) | RERI:-0.828(-1.675,0.019) |
| AT+TT | CT＋TT | 259 | 158 | 0.2 | 0.309 | 1.222(0.831,1.796) | AP:-0.639(-1.303,0.025) |
| AT+TT | CC | 153 | 92 | 0.259 | 0.596 | 1.296(0.497,3.381) | S:0.263(0.071,0.981) |
| rs33002 | rs863221 |  |  |  |  |  |  |
| AA | TG＋GG | 155 | 77 |  |  |  |  |
| AA | TT | 104 | 88 | 0.151 | 0.802 | 1.163(0.357,3.784) | RERI:-0.282(-0.885,0.320) |
| AT+TT | TG＋GG | 251 | 153 | 0.172 | 0.392 | 1.187(0.802,1.759) | AP:-0.264(-0.827,0.298) |
| AT+TT | TT | 160 | 96 | 0.066 | 0.894 | 1.068(0.406,2.810) | S:0.195(0.002,24.120) |
| rs33002 | rs12999145 |  |  |  |  |  |  |
| AA | AA＋AG | 216 | 132 |  |  |  |  |
| AA | GG | 49 | 34 | 0.186 | 0.587 | 1.204(0.616,2.355) | RERI:0.137(-0.629,0.903) |
| AT+TT | AA＋AG | 323 | 193 | 0.126 | 0.535 | 1.134(0.762,1.687) | AP:0.093(-0.413,0.599) |
| AT+TT | GG | 91 | 60 | 0.389 | 0.223 | 1.476(0.789,2.763) | S:1.404(0.169,11.644) |
| rs33002 | rs1981928 |  |  |  |  |  |  |
| AA | AA | 97 | 68 |  |  |  |  |
| AA | TT＋TA | 166 | 98 | 0.167 | 0.544 | 1.182(0.689,2.028) | RERI:0.002(-0.582,0.586) |
| AT+TT | AA | 157 | 95 | 0.155 | 0.566 | 1.168(0.688,1.982) | AP:0.001(-0.431,0.433) |
| AT+TT | TT＋TA | 258 | 157 | 0.301 | 0.290 | 1.351(0.774,2.359) | S:1.005(0.189,5.350) |
| rs33002 | rs4952887 |  |  |  |  |  |  |
| AA | CT＋TT | 86 | 39 |  |  |  |  |
| AA | CC | 178 | 127 | 0.396 | 0.150 | 1.486(0.866,2.549) | RERI:-0.396(-1.222,0.429) |
| AT+TT | CT＋TT | 115 | 65 | 0.389 | 0.208 | 1.476(0.806,2.704) | AP:-0.253(-0.746,0.240) |
| AT+TT | CC | 297 | 186 | 0.448 | 0.121 | 1.566(0.888,2.761) | S:0.588(0.267,1.296) |
| rs33002 | rs2303428 |  |  |  |  |  |  |
| AA | CC | 22 | 14 |  |  |  |  |
| AA | TT＋TC | 240 | 151 | 0.331 | 0.478 | 1.392(0.558,3.471) | RERI:0.088(-0.811,0.987) |
| AT+TT | CC | 48 | 27 | 0.119 | 0.821 | 1.127(0.401,3.166) | AP:0.055(-0.528,0.637) |
| AT+TT | TT＋TC | 367 | 225 | 0.474 | 0.323 | 1.606(0.627,4.115) | S:1.169(0.169,8.069) |
| rs1042821 | rs181747 |  |  |  |  |  |  |
| GG | TT＋TC | 353 | 208 |  |  |  |  |
| GG | CC | 217 | 127 | -0.171 | 0.301 | 0.843(0.609,1.166) | RERI:-0.114(-0.904,0.675) |
| GA+AA | TT＋TC | 60 | 51 | 0.327 | 0.309 | 1.386(0.739,2.601) | AP:-0.103(-0.841,0.636) |
| GA+AA | CC | 37 | 30 | 0.109 | 0.764 | 1.116(0.546,2.278) | S:0.502(0.003,93.019) |
| rs1042821 | rs26779 |  |  |  |  |  |  |
| GG | CT＋TT | 267 | 146 |  |  |  |  |
| GG | CC | 154 | 94 | -0.017 | 0.931 | 0.984(0.674,1.434) | RERI:-0.542(-1.195,0.111) |
| GA+AA | CT＋TT | 147 | 111 | 0.481 | 0.286 | 1.618(0.668,3.921) | AP:-0.512(-1.188,0.164) |
| GA+AA | CC | 100 | 62 | 0.057 | 0.904 | 1.058(0.423,2.646) | S:0.098(0.000,59.324) |
| rs1042821 | rs4952887 |  |  |  |  |  |  |
| GG | CT＋TT | 118 | 74 |  |  |  |  |
| GG | CC | 83 | 30 | -0.797 | **0.007** | 0.451(0.253,0.802) | RERI:0.594(0.243,0.945) |
| GA+AA | CT＋TT | 297 | 185 | -0.116 | 0.601 | 0.891(0.577,1.375) | AP:0.635(0.217,1.054) |
| GA+AA | CC | 171 | 127 | -0.067 | 0.778 | 0.935(0.586,1.492) | S:0.098(0.000,13.645) |
| rs1042821 | rs2303428 |  |  |  |  |  |  |
| GG | CC | 48 | 23 |  |  |  |  |
| GG | TT＋TC | 20 | 16 | 0.824 | 0.102 | 2.279(0.848,6.11) | RERI:-1.773(-3.863,0.317) |
| GA+AA | CC | 365 | 234 | 0.712 | **0.042** | 2.038(1.025,4.051) | AP:-1.147(-2.291,-0.004) |
| GA+AA | TT＋TC | 238 | 142 | 0.435 | 0.220 | 1.545(0.771,3.098) | S:0.235(0.085,0.653) |
| Abbreviations: CHB, chronic hepatitis B; HCC, hepatocellular carcinoma; RERI, Relative Excess Risk of Interaction; AP, Attributable Proportion of interaction; S, Synergy index. When calculating covariance matrix, take SNPs other than the analysis SNPs, together with gender, age, the history of drinking and smoking as control variables. The bold font shows statistical significance. | | | | | | | |

| Supplementary table 11 : The negative results of additive interaction analysis of SNPs between LC and HCC group. | | | | | | | |
| --- | --- | --- | --- | --- | --- | --- | --- |
| SNP1 | SNP2 | LC | HCC | B | *P* | OR(95%CI) | RERI/AP/S |
| rs181747 | rs32950 |  |  |  |  |  |  |
| TT＋TC | AA＋AG | 380 | 225 |  |  | 1.0 |  |
| TT＋TC | GG | 186 | 103 | 0.108 | 0.521 | 1.114(0.801,1.550) | RERI:-2.503(-6.286,1.281) |
| CC | AA＋AG | 7 | 14 | 1.403 | **0.007** | 4.067(1.475,11.214) | AP:-1.491(-3.847,0.856) |
| CC | GG | 77 | 64 | 0.518 | **0.012** | 1.679(1.119,2.517) | S:0.213(0.051,0.893) |
| rs181747 | rs12999145 |  |  |  |  |  |  |
| TT＋TC | AA＋AG | 469 | 256 |  |  | 1.0 |  |
| TT＋TC | GG | 117 | 80 | 0.307 | 0.177 | 1.360(0.870,2.126) | RERI:-0.829(-2.148,0.490) |
| CC | AA＋AG | 69 | 67 | 0.685 | **0.002** | 1.983(1.287,3.054) | AP:-0.547(-1.725,0.631) |
| CC | GG | 18 | 15 | 0.415 | 0.327 | 1.515(0.660,3.476) | S:0.383(0.047,3.115) |
| rs181747 | rs1981928 |  |  |  |  |  |  |
| TT＋TC | AA | 226 | 132 |  |  | 1.0 |  |
| TT＋TC | TT＋TA | 360 | 204 | 0.171 | 0.411 | 1.186(0.790,1.780) | RERI:-0.307(-1.658,1.044) |
| CC | AA | 25 | 32 | 0.726 | **0.026** | 2.066(1.092,3.908) | AP:-0.158(-0.880,0.565) |
| CC | TT＋TA | 62 | 50 62 | 0.666 | **0.021** | 1.947(1.108,3.422) | S:0.775(0.233,2.449) |
| rs181747 | rs4952887 |  |  |  |  |  |  |
| TT＋TC | CT＋TT | 178 | 81 |  |  | 1.0 |  |
| TT＋TC | CC | 408 | 255 | 0.226 | 0.227 | 1.254(0.869,1.810) | RERI:0.049(-1.274,1.372) |
| CC | CT＋TT | 30 | 23 | 0.626 | 0.062 | 1.870(0.970,3.606) | AP:0.023(-0.583,0.628) |
| CC | CC | 57 | 59 | 0.776 | **0.004** | 2.173(1.274,3.706) | S:1.044(0.326,3.344) |
| rs181747 | rs2303428 |  |  |  |  |  |  |
| TT＋TC | TT | 269 | 142 |  |  | 1.0 |  |
| TT＋TC | TC＋CC | 317 | 194 | 0.061 | 0.701 | 1.063(0.778,1.452) | RERI:0.076(-1.062,1.214) |
| CC | TT | 44 | 39 | 0.566 | **0.039** | 1.762(1.030,3.015) | AP:0.040(-0.548,0.628) |
| CC | TC＋CC | 43 | 43 | 0.642 | **0.022** | 1.900(1.099,3.285) | S:1.092(0.289,4.134) |
| rs32950 | rs26279 |  |  |  |  |  |  |
| AA＋AG | GA＋AA | 372 | 224 |  |  | 1.0 |  |
| GG | GG | 36 | 24 | -0.218 | 0.519 | 0.804(0.415,1.558) | RERI:-0.818(-1.381,-0.255) |
| AA＋AG | GA＋AA | 268 | 171 | 0.014 | 0.931 | 1.014(0.741,1.388) | AP:-318260482.928(-537325863.485,-99195102.371) |
| GG | GG | 1 | 0 | -19.779 | 1.000 | 0.000(0.000, ) | S:5.501(0.248,121.912) |
| rs32950 | rs12999145 |  |  |  |  |  |  |
| AA＋AG | AA＋AG | 327 | 186 |  |  | 1.0 |  |
| GG | GG | 81 | 61 | 0.225 | 0.378 | 1.252(0.760,2.062) | RERI:-0.012(-0.764,0.741) |
| AA＋AG | AA＋AG | 218 | 139 | 0.014 | 0.938 | 1.014(0.718,1.431) | AP:-0.009(-0.611,0.593) |
| GG | GG | 51 | 33 | 0.227 | 0.457 | 1.255(0.690,2.283) | S:0.956(0.053,17.102) |
| rs32950 | rs1981928 |  |  |  |  |  |  |
| AA＋AG | AA | 165 | 97 |  |  | 1.0 |  |
| GG | TT＋TA | 243 | 150 | 0.292 | 0.205 | 1.339(0.853,2.103) | RERI:-0.454(-1.135,0.227) |
| AA＋AG | AA | 96 | 71 | 0.247 | 0.295 | 1.280(0.807,2.031) | AP:-0.390(-0.971,0.192) |
| GG | TT＋TA | 173 | 101 | 0.153 | 0.554 | 1.166(0.702,1.935) | S:0.267(0.036,1.991) |
| rs32950 | rs4952887 |  |  |  |  |  |  |
| AA＋AG | CT＋TT | 138 | 62 |  |  | 1.0 |  |
| GG | CC | 270 | 185 | 0.337 | 0.117 | 1.401(0.919,2.137) | RERI:-0.352(-1.089,0.385) |
| AA＋AG | CT＋TT | 87 | 52 | 0.229 | 0.401 | 1.257(0.736,2.146) | AP:-0.270(-0.820,0.280) |
| GG | CC | 182 | 120 | 0.267 | 0.269 | 1.306(0.814,2.095) | S:0.465(0.131,1.650) |
| rs32950 | rs2303428 |  |  |  |  |  |  |
| AA＋AG | TT | 196 | 110 |  |  | 1.0 |  |
| GG | TC＋CC | 212 | 137 | 0.039 | 0.833 | 1.040(0.724,1.493) | RERI:0.063(-0.449,0.575) |
| AA＋AG | TT | 129 | 78 | -0.023 | 0.919 | 0.978(0.632,1.513) | AP:0.058(-0.414,0.530) |
| GG | TC＋CC | 140 | 94 | 0.077 | 0.720 | 1.080(0.709,1.644) | S:4.699(0, ) |
| rs26779 | rs26279 |  |  |  |  |  |  |
| CC＋CT | GA＋AA | 579 | 344 |  |  | 1.0 |  |
| TT | GG | 9 | 3 | -0.488 | 0.548 | 0.614(0.125,3.011) | RERI:0.099(-1.326,1.525) |
| CC＋CT | GA＋AA | 63 | 53 | 0.56 | 0.275 | 1.751(0.640,4.793) | AP:0.068(-0.885,1.020) |
| TT | GG | 28 | 20 | 0.381 | 0.509 | 1.464(0.473,4.537) | S:1.272(0.033,49.308) |
| rs26779 | rs863221 |  |  |  |  |  |  |
| CC＋CT | TT＋TG | 586 | 345 |  |  | 1.0 |  |
| TT | GG | 2 | 2 | -0.213 | 0.867 | 0.808(0.067,9.771) | RERI:0.033(-2.621,2.687) |
| CC＋CT | TT＋TG | 11 | 9 | 0.560 | 0.315 | 1.751(0.588,5.215) | AP:0.021(-1.645,1.687) |
| TT | GG | 80 | 64 | 0.465 | **0.039** | 1.591(1.025,2.472) | S:1.059(0.009,121.313) |
| rs26779 | rs12999145 |  |  |  |  |  |  |
| CC＋CT | AA＋AG | 469 | 266 |  |  | 1.0 |  |
| TT | GG | 119 | 81 | 0.233 | 0.307 | 1.263(0.807,1.975) | RERI:0.025(-1.585,1.635) |
| CC＋CT | AA＋AG | 73 | 58 | 0.588 | 0.252 | 1.801(0.658,4.927) | AP:0.012(-0.752,0.776) |
| TT | GG | 18 | 15 | 0.736 | 0.264 | 2.087(0.575,7.577) | S:1.023(0.231,4.533) |
| rs26779 | rs1981928 |  |  |  |  |  |  |
| CC＋CT | AA | 216 | 137 |  |  | 1.0 |  |
| TT | TT＋TA | 372 | 210 | 0.119 | 0.568 | 1.126(0.748,1.696) | RERI:0.263(-0.927,1.453) |
| CC＋CT | AA | 40 | 29 | 0.510 | 0.384 | 1.666(0.528,5.259) | AP:0.128(-0.422,0.677） |
| TT | TT＋TA | 51 | 44 | 0.720 | 0.188 | 2.054(0.703,6.001) | S:1.332（0.340,5.210） |
| rs26779 | rs4952887 |  |  |  |  |  |  |
| CC＋CT | CT＋TT | 184 | 83 |  |  | 1.0 |  |
| TT | CC | 404 | 264 | 0.272 | 0.142 | 1.312(0.913,1.887) | RERI:-0.518（-2.112,1.076） |
| CC＋CT | CT＋TT | 28 | 23 | 0.839 | 0.152 | 2.313(0.733,7.296) | AP:-0.246（-1.038,0.547） |
| TT | CC | 63 | 50 | 0.746 | 0.167 | 2.110(0.731,6.085) | S:0.681（0.230,2.016） |
| rs26779 | rs2303428 |  |  |  |  |  |  |
| CC＋CT | TT | 267 | 152 |  |  | 1.0 |  |
| TT | TC＋CC | 321 | 195 | -0.014 | 0.933 | 0.987(0.721,1.351) | RERI:0.853(-0.396,2.102) |
| CC＋CT | TT | 49 | 31 | 0.420 | 0.429 | 1.522(0.538,4.308) | AP:0.361(-0.060,0.783) |
| TT | TC＋CC | 42 | 42 | 0.859 | 0.130 | 2.361(0.776,7.181) | S:2.678(0.473,15.181) |
| rs26779 | rs1042821 |  |  |  |  |  |  |
| CC＋CT | GG＋GA | 554 | 325 |  |  | 1.0 |  |
| TT | AA | 34 | 22 | 0.125 | 0.715 | 1.133(0.580,2.216) | RERI:2.118(-2.927,7.162) |
| CC＋CT | GG＋GA | 86 | 67 | 0.563 | 0.272 | 1.756(0.644,4.789) | AP:0.529(-0.096,1.153) |
| TT | AA | 5 | 6 | 1.388 | 0.132 | 4.008(0.657,24.446) | S:3.382(0.487,23.470) |
| rs26279 | rs863221 |  |  |  |  |  |  |
| GA＋AA | TT＋TG | 583 | 349 |  |  | 1.0 |  |
| GG | GG | 59 | 47 | -0.124 | 0.820 | 0.884(0.304,2.567) | RERI:0.055(-0.988,1.098) |
| GA＋AA | TT＋TG | 12 | 6 | -0.265 | 0.684 | 0.767(0.214,2.752) | AP:0.078(-1.383,1.539) |
| GG | GG | 25 | 18 | -0.349 | 0.568 | 0.705(0.212,2.342) | S:0.843(0.039,18.354） |
| rs26279 | rs2303428 |  |  |  |  |  |  |
| GA＋AA | TT | 302 | 171 |  |  | 1.0 |  |
| GG | TC＋CC | 342 | 227 | 0.073 | 0.628 | 1.075(0.801,1.444) | RERI:-0.166(-1.090,0.758) |
| GA＋AA | TT | 16 | 12 | -0.143 | 0.752 | 0.867(0.357,2.104) | AP:-0.214(-1.499,1.071) |
| GG | TC＋CC | 20 | 11 | -0.253 | 0.579 | 0.777(0.318,1.898) | S:3.886(0,2956278.690) |
| rs863221 | rs4952887 |  |  |  |  |  |  |
| TT＋TG | CT＋TT | 195 | 89 |  |  | 1.0 |  |
| GG | CC | 403 | 266 | 0.235 | 0.200 | 1.265(0.883,1.813) | RERI:-0.184(-1.009,0.641) |
| TT＋TG | CT＋TT | 25 | 19 | 0.002 | 0.998 | 1.002(0.305,3.285) | AP:-0.170(-0.952,0.613) |
| GG | CC | 57 | 47 | 0.08 | 0.884 | 1.084(0.367,3.199) | S:0.312(0.001,93.159) |
| rs12999145 | rs1981928 |  |  |  |  |  |  |
| AA＋AG | AA | 125 | 74 |  |  | 1.0 |  |
| GG | TT＋TA | 421 | 252 | 0.108 | 0.592 | 1.114(0.751,1.653) | RERI:1. 812(-5.067,8.691) |
| AA＋AG | AA | 133 | 91 | 0.188 | 0.396 | 1.207(0.782,1.863) | AP:0.578(-0.357,1.514) |
| GG | TT＋TA | 1 | 4 | 1.142 | 0.337 | 3.132(0.304,32.257) | S:6.647(0.148,298.642) |
| rs12999145 | rs2303428 |  |  |  |  |  |  |
| AA＋AG | TT | 299 | 169 |  |  | 1.0 |  |
| GG | TC＋CC | 245 | 157 | 0.088 | 0.572 | 1.092(0.804,1.483) | RERI:-0.281(-1.465,0.904) |
| AA＋AG | TT | 18 | 13 | 0.408 | 0.366 | 1.504(0.621,3.641) | AP:-0.213(-1.128,0.701) |
| GG | TC＋CC | 118 | 82 | 0.274 | 0.262 | 1.315(0.815,2.123) | S:0.529(0.059,4.734) |
| rs12999145 | rs1042821 |  |  |  |  |  |  |
| AA＋AG | GG＋GA | 513 | 307 |  |  | 1.0 |  |
| GG | AA | 31 | 19 | 0.009 | 0.980 | 1.009(0.511,1.992) | RERI:3.713(-1.975,9.401) |
| AA＋AG | GG＋GA | 128 | 86 | 0.171 | 0.437 | 1.186(0.771,1.824) | AP:0.756(0.443,1.070) |
| GG | AA | 8 | 9 | 1.591 | 0.062 | 4.908(0.924,26.063) | S:19.990(0.360,1108.877) |
| rs1981928 | rs1042821 |  |  |  |  |  |  |
| AA | GG＋GA | 242 | 152 |  |  | 1.0 |  |
| TT＋TA | AA | 14 | 13 | 0.935 | 0.125 | 2.547(0.772,8.401) | RERI:-1.574(-4.052,0.903) |
| AA | GG＋GA | 399 | 241 | 0.173 | 0.387 | 1.189(0.803,1.761) | AP:-1.355(-3.856,1.146) |
| TT＋TA | AA | 25 | 15 | 0.15 | 0.706 | 1.162(0.532,2.538) | S:0.093(0.001,12.775) |
| rs4952887 | rs2303428 |  |  |  |  |  |  |
| CT＋TT | TT | 139 | 65 |  |  | 1.0 |  |
| CC | TC＋CC | 74 | 41 | 0.164 | 0.536 | 1.178(0.701,1.982) | RERI:-0.153(-0.845,0.539) |
| CT＋TT | TT | 181 | 121 | 0.269 | 0.207 | 1.308(0.862,1.985) | AP:-0.115(-0.626,0.396) |
| CC | TC＋CC | 286 | 194 | 0.288 | 0.191 | 1.333(0.866,2.054) | S:0.686(0.171,2.748) |
| rs4952887 | rs1042821 |  |  |  |  |  |  |
| CT＋TT | GG＋GA | 199 | 100 |  |  | 1.0 |  |
| CC | AA | 13 | 5 | -0.213 | 0.734 | 0.808(0.237,2.757) | RERI:0.790(-0.639,2.219) |
| CT＋TT | GG＋GA | 442 | 293 | 0.18 | 0.309 | 1.197(0.847,1.691) | AP:0.440(-0.197,1.077) |
| CC | AA | 26 | 23 | 0.585 | 0.127 | 1.796(0.847,3.806) | S:147.947(0,) |
| rs2303428 | rs1042821 |  |  |  |  |  |  |
| TT | GG＋GA | 299 | 172 |  |  | 1.0 |  |
| TC＋CC | AA | 17 | 11 | 0.589 | 0.209 | 1.803(0.719,4.522) | RERI:-0.822(-2.489,0.846) |
| TT | GG＋GA | 342 | 221 | 0.094 | 0.531 | 1.099(0.818,1.476) | AP:-0.762(-2.580,1.057) |
| TC＋CC | AA | 22 | 17 | 0.076 | 0.855 | 1.079(0.479,2.431) | S:0.088(0.000,1415.921) |
| Abbreviations: LC, liver cirrhosis; HCC, hepatocellular carcinoma; RERI, Relative Excess Risk of Interaction; AP, Attributable Proportion of interaction; S, Synergy index. When calculating covariance matrix, take SNPs other than the analysis SNPs, together with gender, age, the history of drinking and smoking as control variables. The bold font shows statistical significance. | | | | | | | |

**2 SUPPLEMENTARY FIGURES**


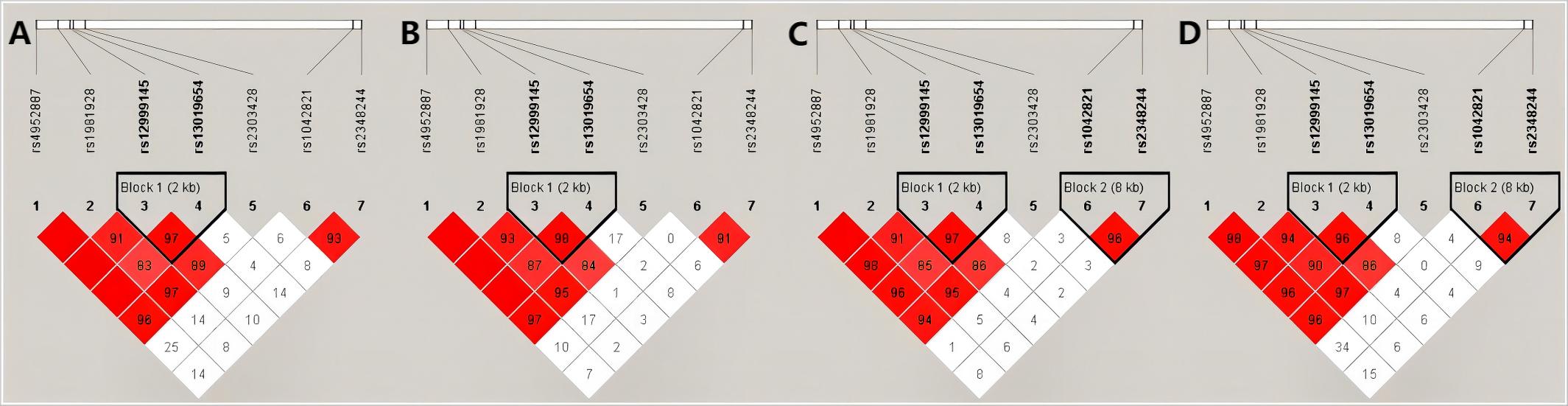


**Figure S1.** Haplotype analysis for 7 SNPs near *MSH2*, *MSH6* gene in HC vs. HCC(**A**), NC vs. HCC(**B**), CHB vs. HCC(**C**), and LC vs HCC(**D**) groups by Haploview 4.2

.


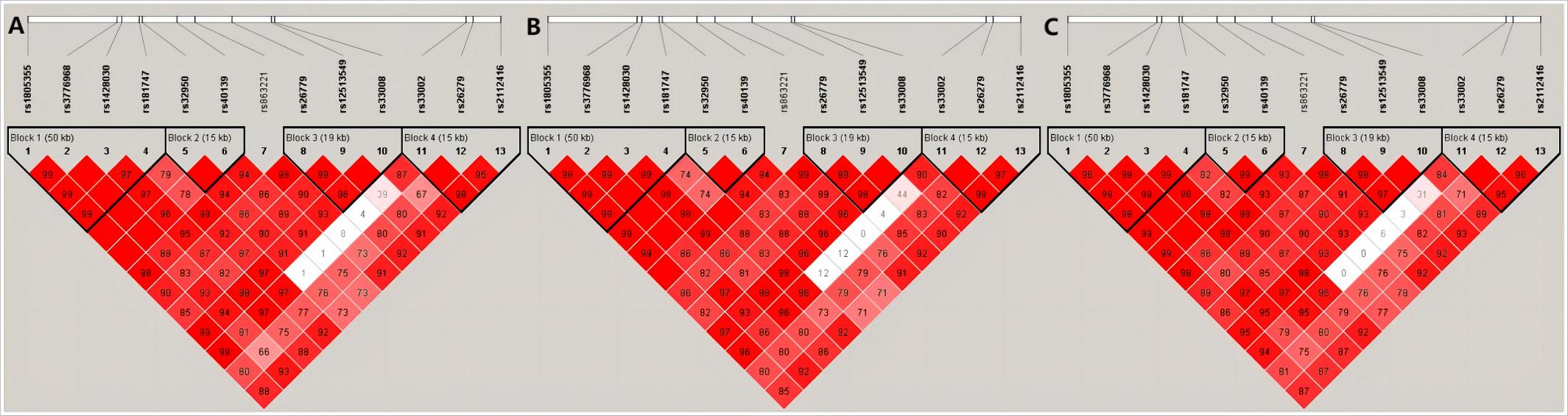


**Figure S2.** Haplotype analysis for 13 SNPs near MSH3 gene in NC vs. HCC(**A**), CHB vs. HCC(**B**), and LC vs HCC(**C**) groups by Haploview 4.2
